# CFD and site analysis for optimizing indoor air quality in sustainable social housing via windcatcher integration

**DOI:** 10.1038/s41598-026-39870-0

**Published:** 2026-03-23

**Authors:** Mohamed Ibrahim Abdelhady, Mohamed I. A. Habba, Mohamed Abdelfadel Alsaber, Asmaa Abd Elmoneim Fahmi

**Affiliations:** 1 Civil and Architectural Constructions Department, Faculty of Technology and Education, Suez University, P. O. Box: 43221, Suez, Egypt; 2 Mechanical Department, Faculty of Technology and Education, Suez University, P.O.Box: 43221, Suez, Egypt

**Keywords:** Windcatcher, Egyptian social housing, Natural ventilation, CFD simulation, Natural ventilation, Sustainability, Civil engineering, Environmental impact

## Abstract

This study investigated the potential of integrating windcatchers into Egyptian social housing buildings to enhance natural ventilation and improve indoor thermal comfort, thereby reducing energy consumption used to enhance airflow inside residential spaces of residential communities, which is considered an important aspect of sustainable urban development that aims to counteract climate change. Computational Fluid Dynamics (CFD) simulations validated by site measurements were conducted on two distinctly oriented apartment models. The northwest-oriented apartment demonstrated a 7% improvement in natural ventilation rate with a single strategically placed windcatcher in the bedroom. The southwest-oriented apartment showed a remarkable 45.7% improvement when equipped with three windcatchers distributed across the living room and bedroom spaces. The results revealed that strategic windcatcher placement was more critical than quantity, with a dual-windcatcher configuration in the southwest-oriented apartment achieving a performance nearly comparable to that of a triple-windcatcher arrangement. Effectiveness relies on establishing appropriate pressure gradients through careful positioning to facilitate consistent airflow circulation. Configurations that enhance living room ventilation, the primary family gathering space, demonstrated the greatest potential for improving overall thermal comfort. Practical recommendations include orientation-specific design strategies, early integration of windcatchers into the architectural design process, and standardization of building codes for social housing projects. The implementation of passive cooling strategies offers multiple benefits, including reduced reliance on mechanical cooling, improved indoor air quality, connection to architectural heritage, and significant cumulative energy savings at scale. Future research directions encompass seasonal performance studies, combinations with other cooling strategies, and detailed analyses of windcatcher geometry.

## Introduction

Buildings consume approximately 40% of the global energy resources., which means they will be crucial in lowering global energy consumption, which means they will be crucial in lowering global energy consumption^1^, and the percentage is predicted to rise up to 50 percent by 2050^1^. Cooling and ventilation are major factors contributing to high building energy consumption, especially in tropical and hot regions. As a result, researchers in almost all specializations are seeking solutions to reduce the energy requirements of ventilation and cooling in buildings. Many works^2–4^ have shown that natural ventilation and passive cooling strategies can reduce energy consumption during the cooling season and, at the same time, improve the indoor air quality and, in some cases, the comfort level of occupants.

Numerous studies have demonstrated that natural ventilation and passive cooling solutions can reduce energy consumption during the cooling season while also improving indoor air quality and, in some situations, occupants’ comfort^5^.

Windcatchers have long been used in traditional Middle Eastern architecture and recently, a modern form of the windcatcher was used in buildings in the United Kingdom, including schools and open-space offices^6–8^. The proposed design solutions for integrating windcatchers into Egyptian social housing projects will result in significant energy savings and increased sustainability at the national level.

**Fig. 1:**
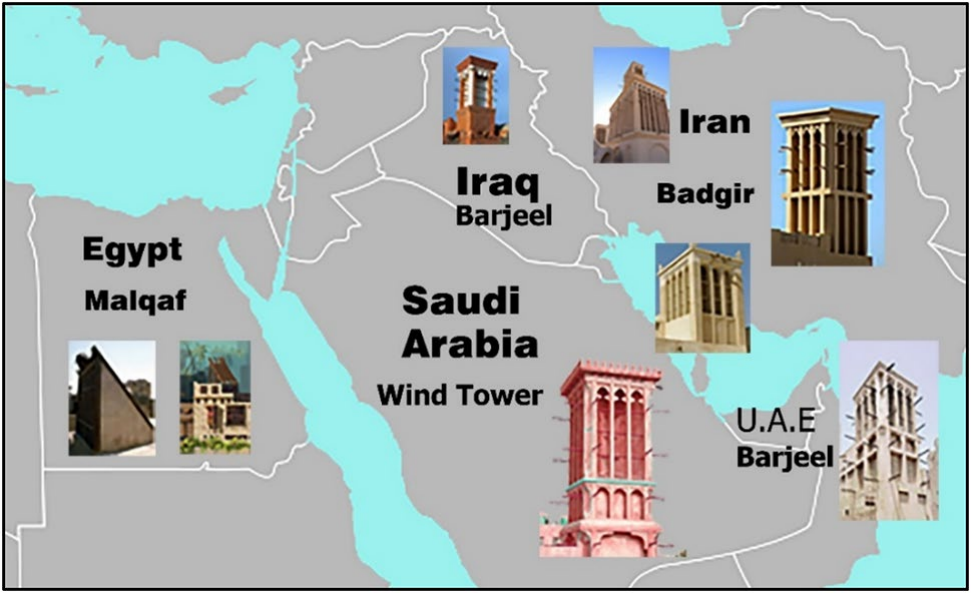
Different Forms of Windcatchers in the Middle East; the map generated by https://dashboard.visme.co/v2/home and modified by Adobe Photoshop 2022 software.

According to the UN, approximately 68% of the world’s population will reside in cities by 2050. These cover only 3% of the Earth’s surface, yet utilize 78% of the energy and emit 60% of the greenhouse gases. In tropical developing countries, the Energy demand grows by a factor of 2–4. Globally, the 2050 baseline energy demand is two to three times larger than it is today for the heating, ventilation, and air conditioning (HVAC) sector, which uses over two-thirds of all energy^9,10^. Building energy consumption is substantial in terms of both its current size and future increases. This offers a significant opportunity to reduce greenhouse gas emissions and energy usage^9^. This problem may be resolved by using less mechanical HVAC; however, it is crucial to maintain indoor air quality and thermal comfort simultaneously. The worldwide epidemic has increased awareness of the significance of indoor air quality, making this problem more pressing than before. The World Health Organization (WHO) indicates that in crowded and poorly ventilated spaces, the risk of getting COVID-19 infection is increased as the infected aerosols can remain suspended in the air or travel farther than conversational distance, so improving indoor ventilation rates reduces the risk of the virus spreading inside functional spaces^10^. Natural ventilation may be very beneficial in buildings with simple planning and few interior impediments, and in regions with moderate or warm weather. When natural ventilation is used, airflow is facilitated by openings such as windows and doors, and the interior airflow is improved by architectural elements such as vents, louvers, passive chimneys, and windcatchers, which were used in ancient Egypt to ventilate indoor air naturally. Middle Eastern regions, including the small Arab states of the Persian Gulf (Fig. 1), such as Afghanistan, Dubai, and Pakistan, continue to use these elements. Windcatchers can be classified into three categories based on the direction in which they capture the wind: multidirectional, two-directional, and unidirectional. They can be further categorized based on their design, specifically whether they employ ascending or descending air currents^11,12^. Windcatchers are designed to utilize descending air currents to compress air and minimize room temperature. The upper section of the tower is positioned at an elevated location, such as a chimney, and is enclosed on all sides except one, which features an open framework designed to capture the wind. The wind entering through the open side generated a downward air current that descended toward the center of the room, resulting in a cooling effect. Some benefits of this method include the elimination of the need for a separate cooling process for incoming air and an improvement in cooling efficiency as the descending air current speed increases^13,14^.

**Fig. 2:**
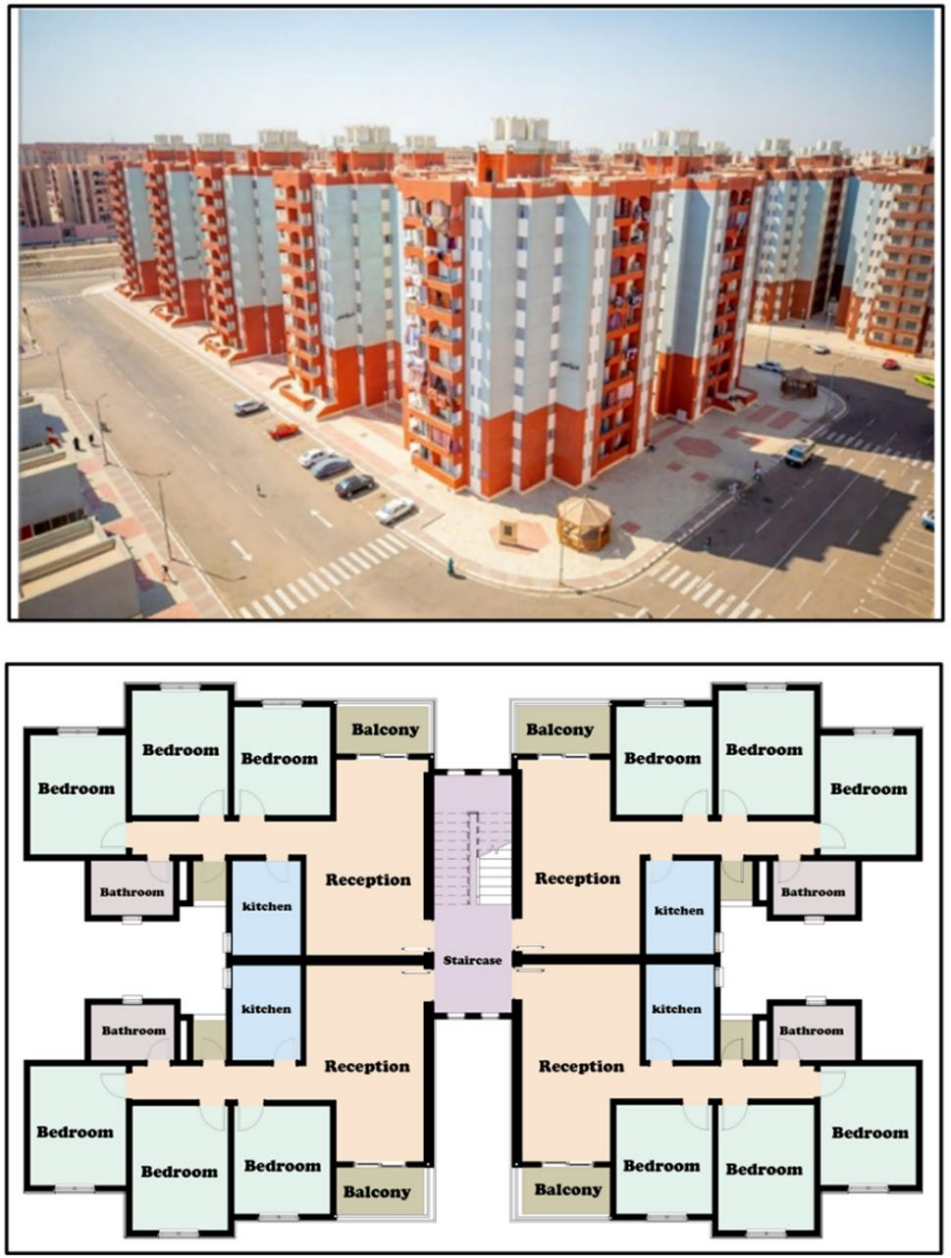
Architectural design of social housing buildings in Egypt.

**Fig. 3:**
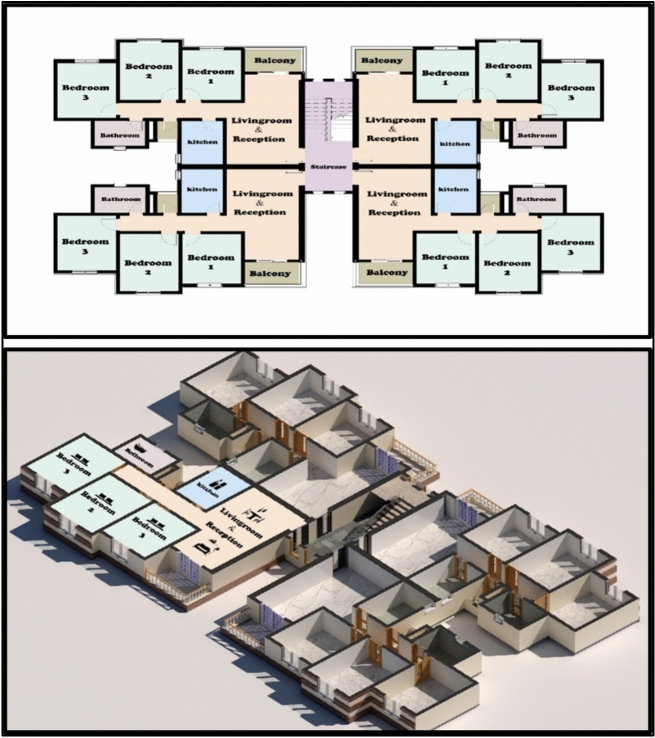
Social housing buildings in Egypt.

Egypt aims to ensure that individuals with low and medium incomes have access to suitable housing options that align with their financial constraints. The New Urban Communities Authority is responsible for proposing, planning, and implementing social housing projects as part of the state’s economic and social development. These projects aim to provide suitable housing for low-income citizens, in line with the social housing program (Figs. 2, 3). The social housing program offers housing accommodations for individuals with restricted income in specific regions. As part of this initiative, the authority proposed housing projects in the social housing program across 22 newly developed cities. Therefore, any proposals to save energy in residential models by improving natural ventilation rates within different residential spaces will lead to significant energy savings and increase sustainability at the state level. Given that a growing proportion of individuals in contemporary society spend up to 80% of their time indoors, the air they inhale must be of high quality^14–17^. The WHO Occupational Safety and Health to about 1.5 million deaths in 2000^18,19^. Additionally, indoor air pollution has been identified as the third most significant contributor to disability-adjusted life years globally^20^.

The National Institute for Occupational Safety and Health in the USA (NIOSH) has reported that inadequate ventilation is the primary cause of 52% of the pollutants detected in indoor air^21^. Using suitable ventilation techniques plays an important role in enhancing the indoor air quality as it helps disperse indoor air pollutants, such as fine particles and bioaerosols, by introducing a fresh inflow of air. This study aims to use wind catchers in social housing buildings to improve thermal comfort inside the building by increasing the rates of natural ventilation, which helps in reducing the energy used in buildings, by studying the effect of the locations and number of wind catchers in social housing buildings and comparing them to choose the best models with the best rates of natural ventilation inside the building. In general, there are three types of ventilation: Firstly, using architectural building elements such as window openings, atria and courtyards, wing walls, chimney cowls, wind towers, and wind catchers^22^. Secondly, mechanical, such as air conditioners and fans^23^, and thirdly: a hybrid type^24^. Because natural ventilation mostly depends on buoyancy and wind forces, it may provide significant energy savings compared to mechanical ventilation. Researchers have shown interest in wind towers and windcatchers as excellent examples of natural ventilation devices. For ages, wind towers have been used in Middle Eastern structures to provide passive ventilation and cooling. Bahadori^25^ examined the ability of a novel wind tower design to provide passive cooling and natural ventilation as early as 1984. The viability of the proposed design was assessed through theoretical calculations. In the revised design, the interior temperature was lowered, ranging between 2.5 °C and 4 °C lower, whereas the supply air velocity increased. The results show that opening the wind tower reduces the inside temperature and improves cooling efficiency compared with closing it. Additionally, there was less dust in the air.

Numerous studies have concentrated on enhancing cooling performance using mechanical means, such as evaporative cooling techniques, instead of using architectural solutions, such as an integrated windcatcher, which help reduce the temperature without using any energy. For instance, Bouchahm et al.^14^ and Mohamed^26^. However, other studies have focused on improving ventilation efficiency using wind tower design optimization. The ventilation effectiveness of wind towers with square, hexagonal, and six-opening circular cross sections was examined by Farouk^27^. The induced airflow volume was primarily determined by the speed of the external wind. Compared to the square windcatcher, the hexagonal windcatcher had a 19% greater air velocity and a roughly 20% lower total air volume. To improve ventilation efficiency, some studies have recently integrated wind towers with other technologies, including solar chimneys^2^, courtyards^28^, and renewable energy systems^29^. Prior research has suggested that windcatchers are extensively employed in areas characterized by wind speeds ranging from 3 to 5 m/s. The utilization and impact of windcatchers are considerably constrained and inconsequential under conditions characterized by lower wind speeds^30^. Montazeri^31^examined various aspects of utilizing wind catchers for cross-ventilation. The study provided a detailed examination of the impact of outlet openings on the ventilation performance of a single-zone isolated building with a wind catcher.

Also, many researchers have specifically examined the utilization of wind catchers as architectural components for enhancing both indoor and outdoor natural ventilation rates^30,32^. Other studies have examined the impacts of integrating wind catchers with other passive ventilation solutions, such as evaporative cooling systems, domes, and solar chimneys, in building design^33,34^. This study aims to investigate the effectiveness of integrating windcatchers into Egyptian social housing buildings to enhance natural ventilation and indoor thermal comfort, thereby reducing the energy consumption associated with mechanical cooling systems.

Utilizing CFD simulations validated by on-site measurements, this study evaluated the impact of windcatcher placement and quantity on airflow rates within residential units oriented differently (northwest and southwest). This study identified the optimal configurations for each orientation. The practical recommendations from the analysis include orientation-specific design strategies and the early integration of windcatchers into architectural planning. Finally, this study aims to promote sustainable urban development by improving indoor environmental quality, reducing reliance on mechanical ventilation systems, connecting contemporary architecture with traditional heritage, and achieving significant cumulative energy savings at a broader scale. The proposed design solutions for integrating windcatchers into Egyptian social housing projects will result in significant energy savings and increased sustainability at the national level.

## Experimental and modeling

### Case study description

**Fig. 4:**
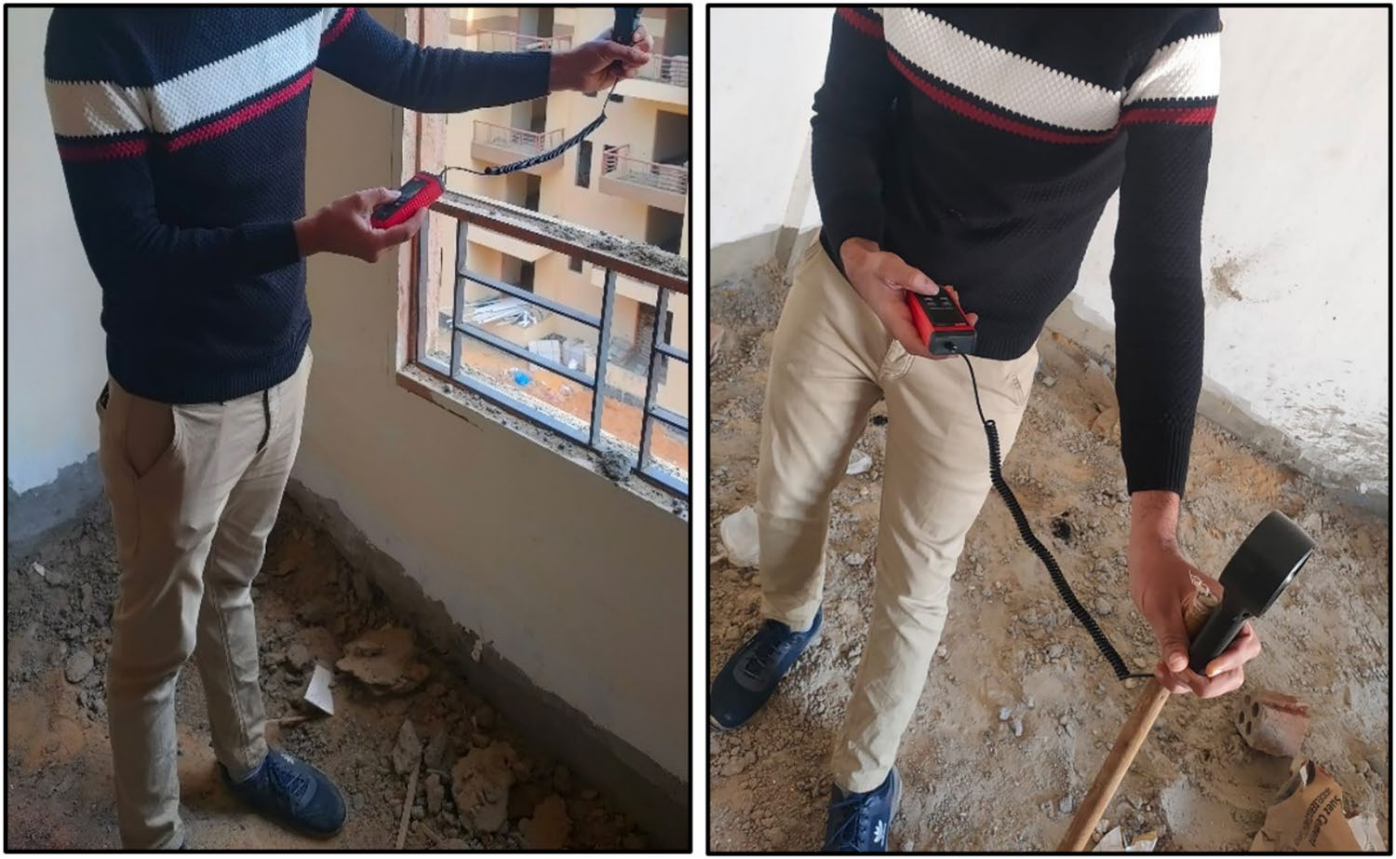
Air speed measurements inside the building.

This study used a social housing building in Egypt as the primary case study. The building consists of a ground floor and five typical floors. The building dimensions are 27.4 m in length, 15.9 m in width, and 18 m in height. The building design does not contain a windcatcher. The methodology commenced with comprehensive field measurements (Fig. 4), which involved collecting wind speed data in each room of the residential building. For each room, measurements were divided into 30 sampling points, and average wind speed values were calculated for validation.

**Fig. 5:**
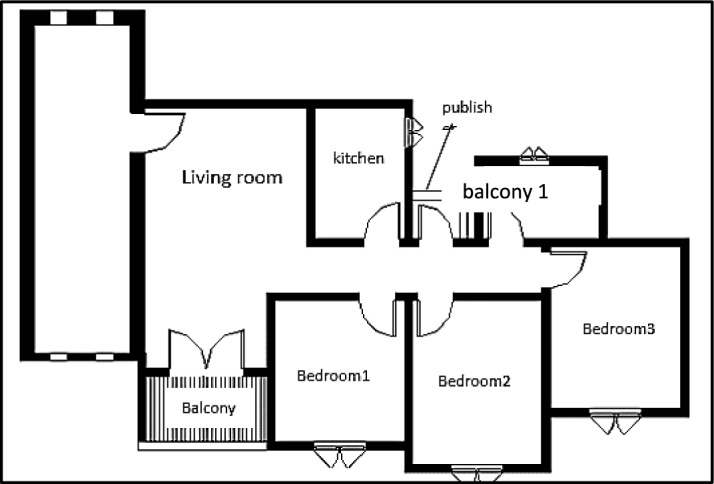
The First Apartment (A).

**Fig. 6:**
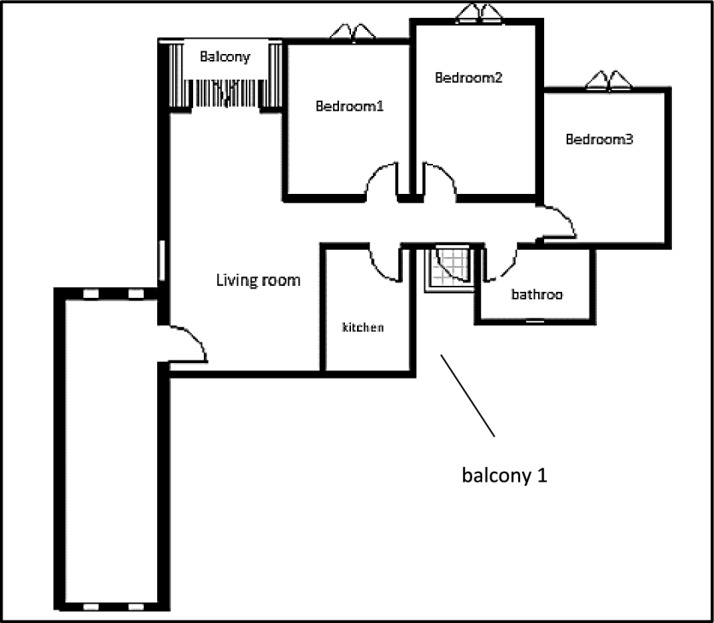
Second Apartment (B).

External wind conditions were measured using two precision instruments, a Testo 440 anemometer and a UNI-T device, to ensure accurate characterization of the ambient wind environment Table 1. Subsequently, detailed three-dimensional models of the two selected apartments within the building were created using Autodesk Revit. The first Apartment A designated as Apartment A, was situated with a northwest orientation (Fig. 5), while the second apartment, designated as Apartment B, had a southwest orientation (Fig. 6). This deliberate selection allowed for the analysis of windcatcher performance in different building orientations under actual environmental conditions.

**Table 1:** Different tools, devices, and software used in site measurements.

Tool/Equipment	Manufacturer	Purpose	Range	Resolution	Accuracy	Country	Photo
Anemometer	Testo 404	To measure air speed using a wire	0.2–30 m/s	0.1 m/s	± (.3m\s + 5%) of reading	Germany	
Compass		Determine the main directions of the building				China	
Meter		Measuring lengths and heights				China	
Anemometer	Uni-T	To measure air speed using wire	0.4–20 m/s	0.1 m/s	± (.3m\s + 5%) of reading	China	
CFD	Autodesk	Predict air pattern inside and outside objects				USA	
Revit Architecture	Autodesk	For building modeling				USA

### Experimental design and windcatcher configuration

Multiple configurations were developed for each apartment model to systematically evaluate the windcatcher integration. For Apartment A (northwest orientation), three distinct windcatcher arrangements were implemented, as detailed in Table 2.

- Case 1: Two windcatchers positioned at balcony 1 and bedroom 3
- Case 2: Single windcatcher positioned at balcony 1
- Case 3: Single windcatcher positioned in bedroom 3

**Table 2:**
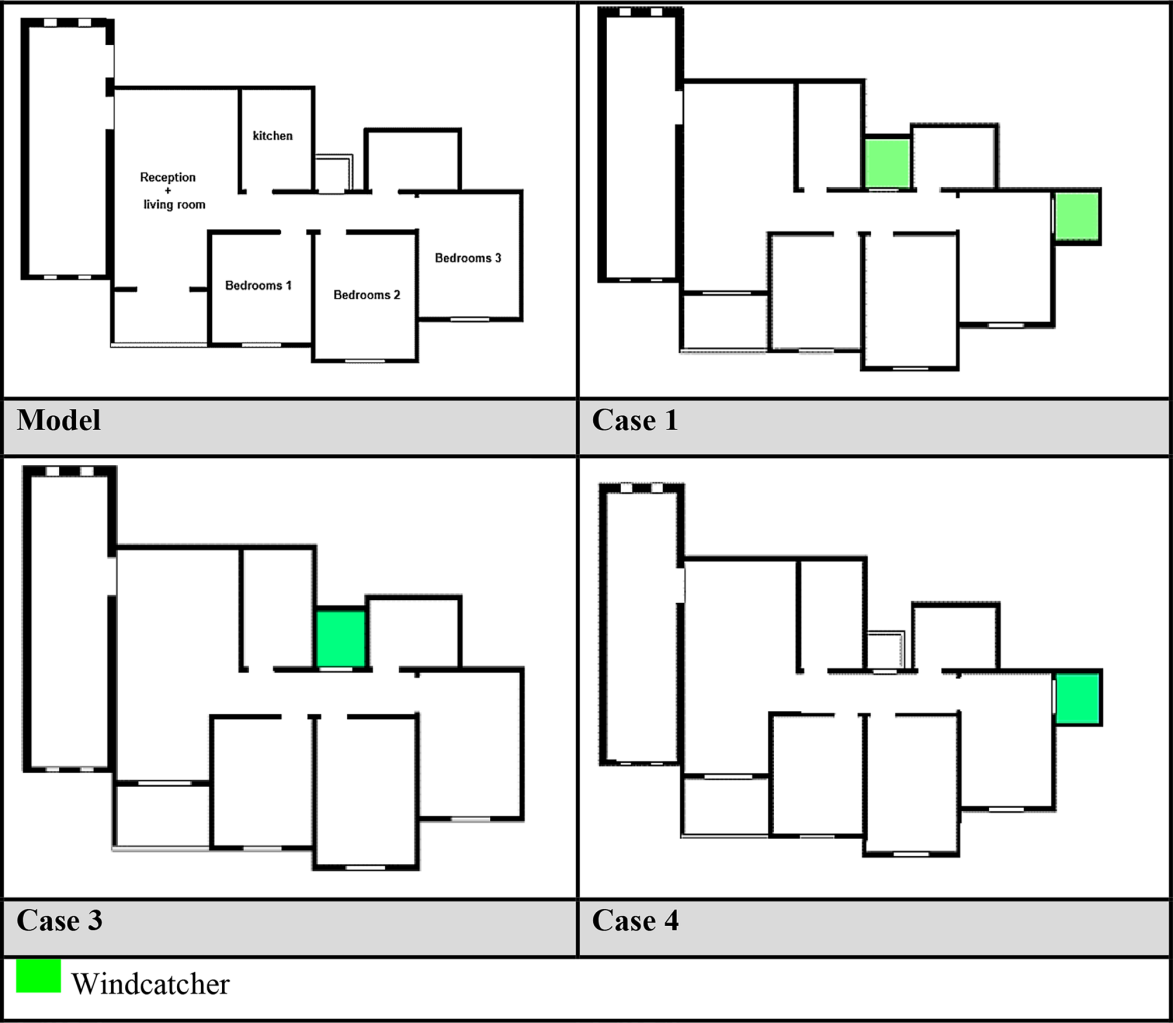
Different cases of windcatchers placement in the first apartment, Northwest Orientation.

For Apartment B (southwest orientation), a more extensive series of seven different configurations was developed, as presented in Table 3.

- Cases 3, 5, and 6: Single windcatcher in different locations
- Cases 1, 4, and 7: Two windcatchers in different locations
- Case 2: Three windcatchers are distributed throughout the apartment

**Table 3:**
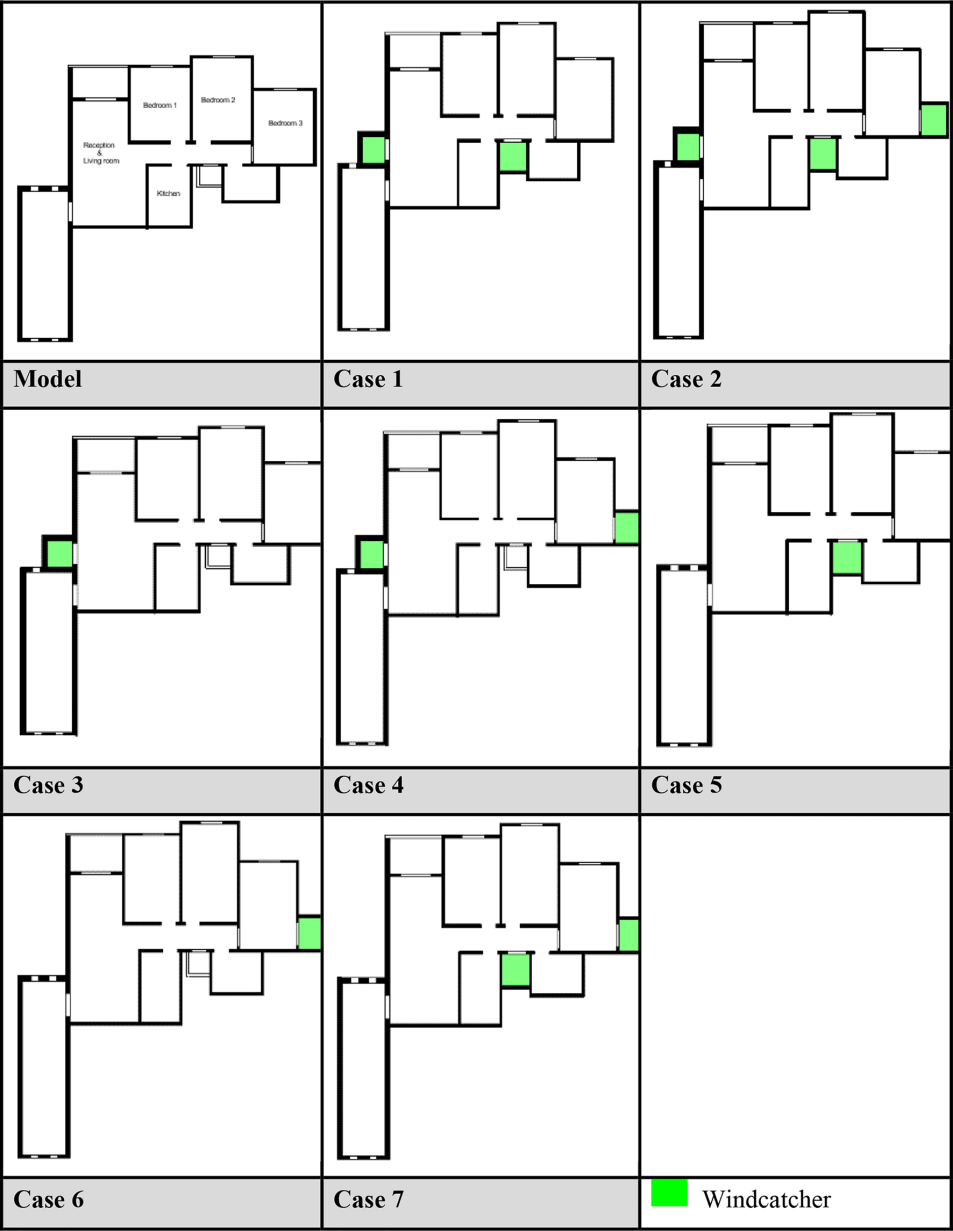
Different cases of windcatchers placement in the second apartment (Southwest orientation).

The windcatcher design consisted of a rectangular cuboid with dimensions of 1.5 m in length, width, and height. In the experimental setup, the windcatcher aperture served as the air inlet, and the apartment openings functioned as outlets, creating a complete ventilation circuit.

### CFD simulation methodology

#### Software and computational domain

In the field of this study, Computational fluid dynamics (CFD) is considered as an extremely useful technique for analyzing the working principles of wind catchers depending on different factors such as the type of wind catcher (number of openings on the tower), the turbulence modeling approach (RANS and LES), turbulence model implemented, the type of inlet velocity profile (uniform and atmospheric boundary layer (ABL) and It can be concluded that: almost numerous studies have demonstrated the CFD studies focus on the followings: (i) wind catchers geometry, (ii) buoyancy-driven ventilation and (iii) the performance of wind catchers integrated with evaporative cooling systems and heat exchangers.^31^.

The main purpose of the previous CFD studies was to analyze the ventilation performance of the wind catcher by focusing on the induced airflow rate, and disregarding the indoor air quality inside the ventilated building. Therefore, this current study investigates the impact of integrated wind catchers (placement and quantity) as inlet openings on basic flow characteristics of cross-ventilation into a single-zone isolated building in a neutral atmospheric boundary layer (ABL). High-resolution coupled (outdoor wind flow and indoor airflow) 3D steady RANS CFD simulations of cross-ventilation are performed for the building model with different locations and quantities. The CFD simulations are validated based on site measurements. The main goal of the CFD simulation is to provide design alternatives for typical social housing buildings, which are built in many Egyptian governorates, in order to increase natural ventilation and cooling rates within functional architectural spaces without the use of mechanical means, resulting in significant energy consumption savings. This study differs from earlier research in that it uses repeated actual multi-story dwelling models.

**Fig. 7:**
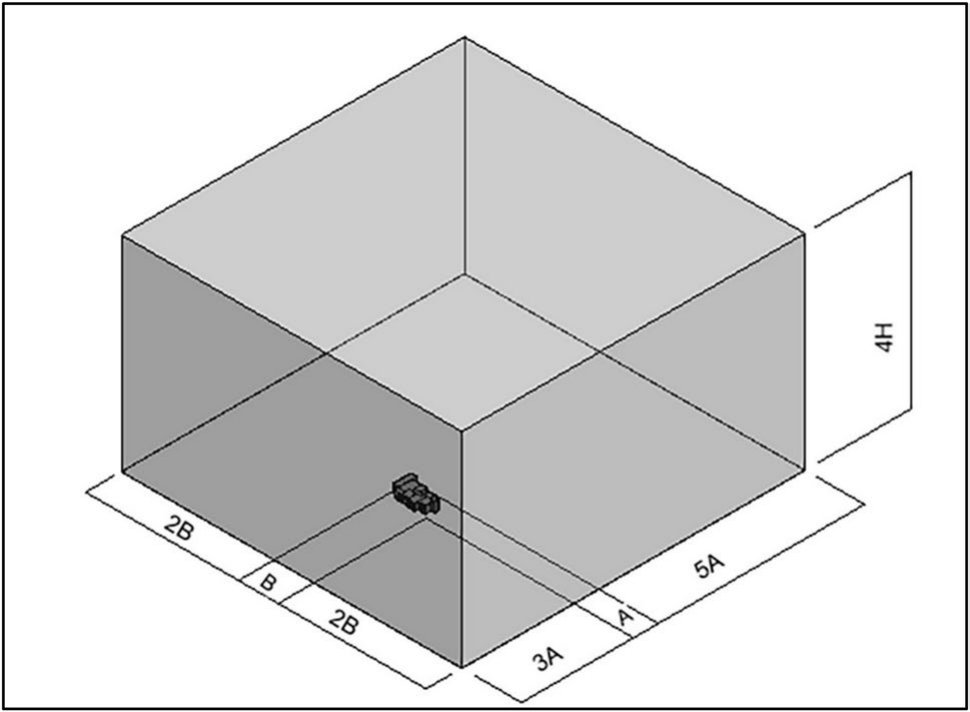
Relation between reference model and external volume.

**Fig. 8:**
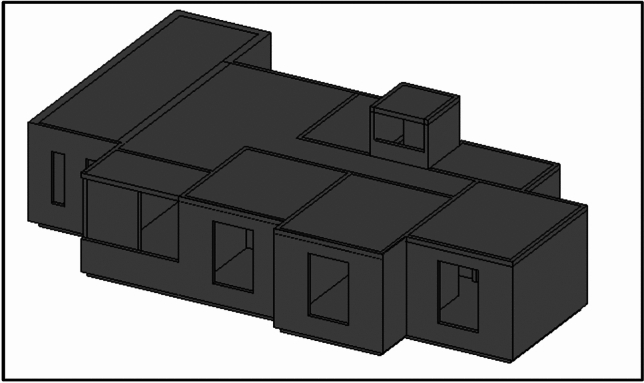
Reference model with windcatcher.

The investigation of windcatcher performance under low wind speed conditions specifically focused on air velocity and airflow distribution. Computational fluid dynamics simulations were performed using Autodesk CFD 2019, which incorporates robust three-dimensional computational capabilities specifically designed for architectural applications. Each model incorporated an external computational domain (External reference domain) sized proportionally to the building dimensions to accurately capture the approaching wind patterns and external airflow dynamics, following established CFD best practices for architectural ventilation studies. The distance between the external reference and the building model depends on the building dimensions, while A refers to building length, B refers to building width, and H refers to building height (Figs. 7, 8).

#### Building materials and boundary conditions

**Fig. 9:**
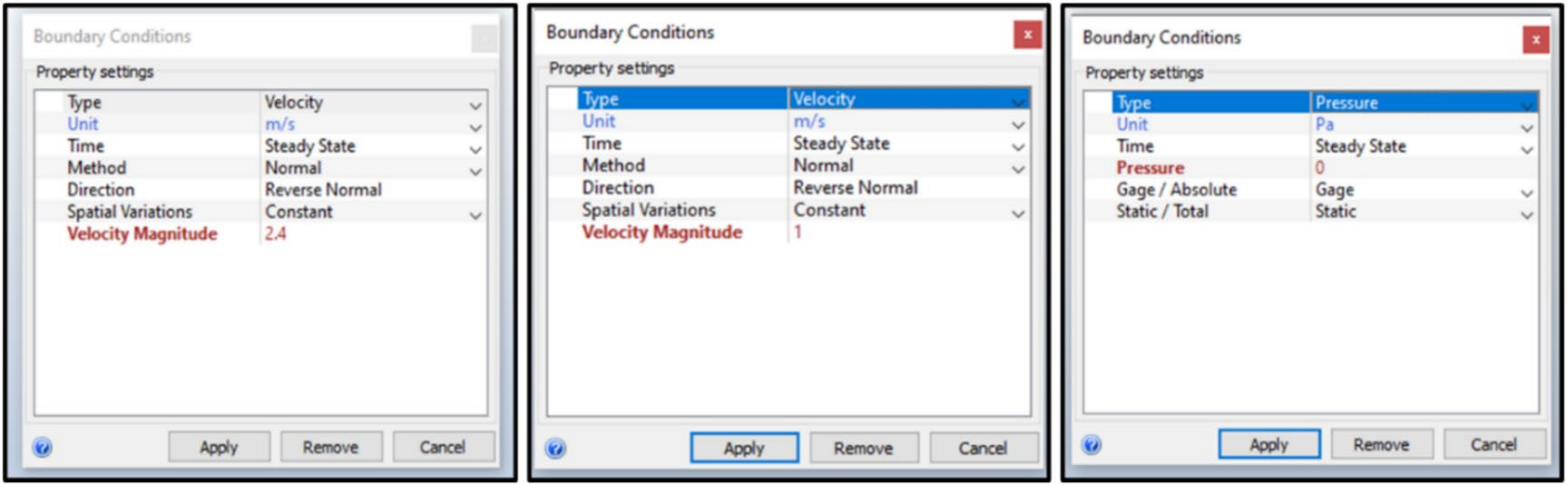
Boundary conditions of the simulation.

The simulation employed realistic construction materials, with windcatcher walls and model walls composed of 25 cm thick bricks, while windcatcher roofs and building floors utilized 20 cm thick concrete. Both the external and internal volumes were populated with natural air under ambient conditions to accurately model the thermal and aerodynamic properties. For Apartment A with a northwest orientation, the boundary conditions incorporated neutral atmospheric boundary layer inflow profiles with a mean wind speed of 2.4 m/s directed toward the northwest facade (Fig. 9). For Apartment B with a southwest orientation, the average approaching wind velocity was 1.0 m/s. The pressure at the domain outlets was maintained at 0 Pa to establish equilibrium conditions.

#### Mesh generation and quality assurance

**Fig. 10:**
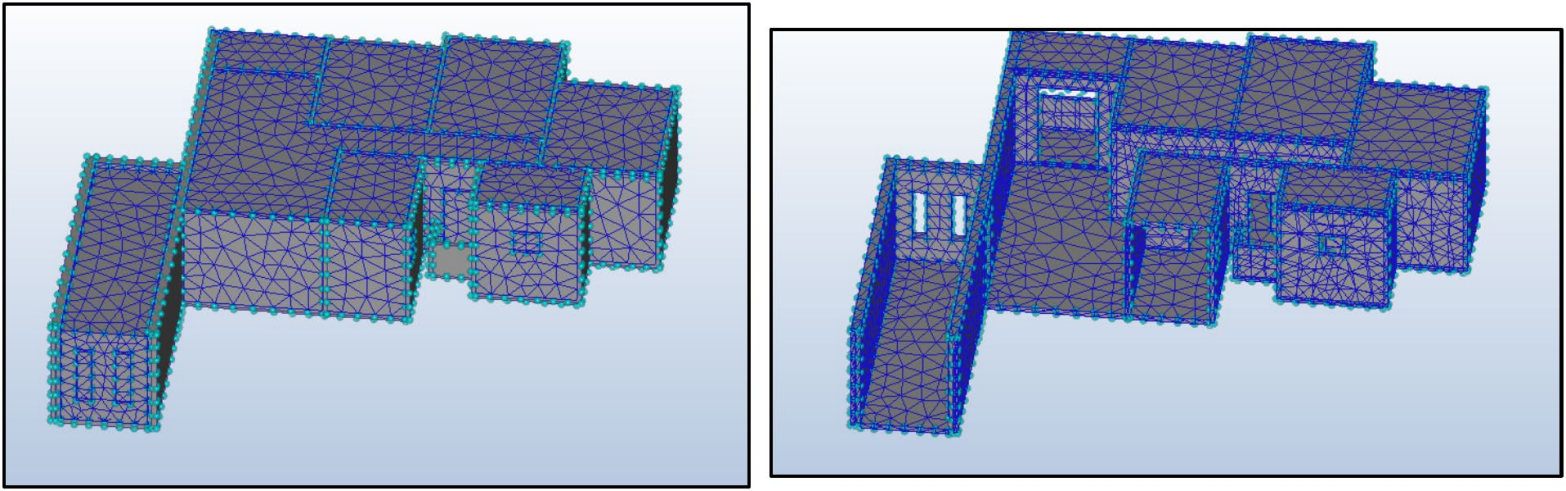
The mesh of the model.

**Fig. 11:**
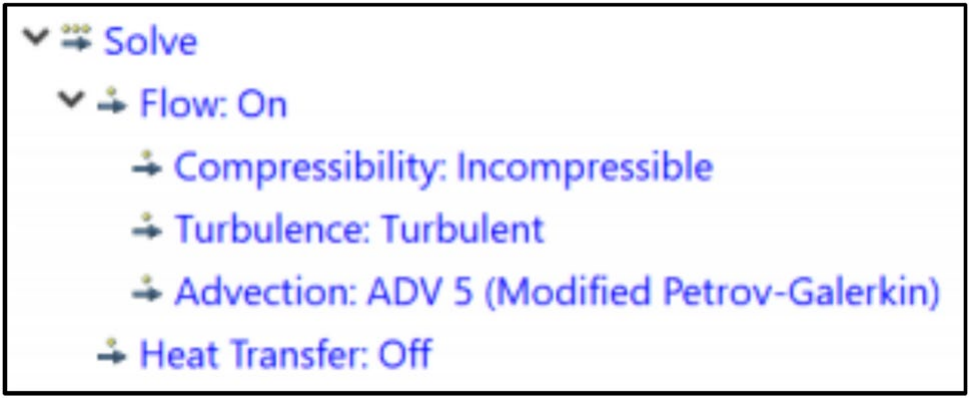
Solve settings.

The meshing process, a critical determinant of simulation accuracy, involved extensive testing of multiple configurations across geometric models. After systematic refinement, a mesh composed exclusively of tetrahedral elements was selected for the optimal resolution of the flow features (Fig. 10). The auto-sizing meshing algorithm implemented in Autodesk CFD performed a comprehensive topological interrogation of the analysis geometry, determining the optimal mesh size and distribution for each edge, surface, and volume within the models (Fig. 11). Mesh discretization followed established guidelines for architectural CFD simulations, with particular attention to refinement near critical flow regions, such as windcatcher inlets, outlets, and areas of expected flow separation. This approach ensured an appropriate resolution of the boundary layers and recirculation zones, which are critical for the accurate prediction of ventilation performance.

#### Solver settings and simulation parameters

**Fig. 12:**
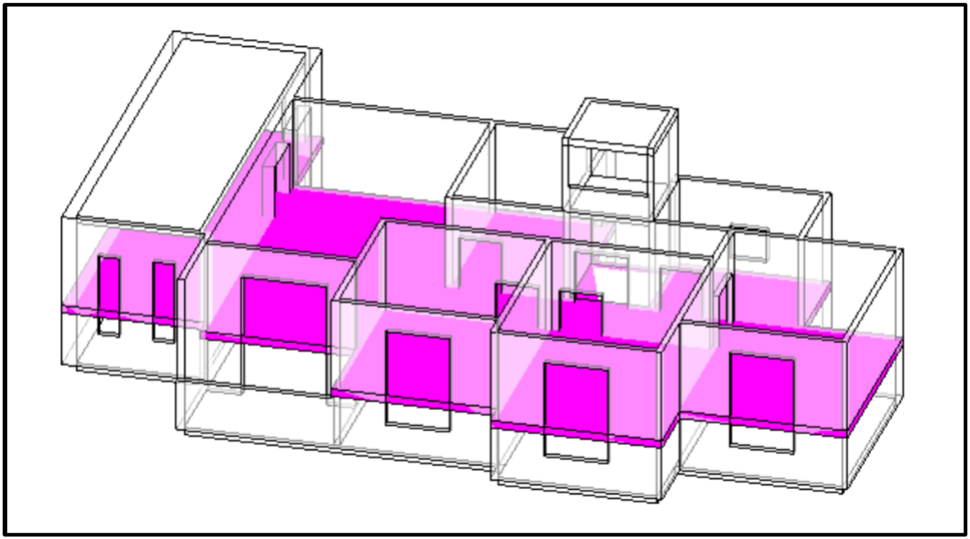
The position of the plane for the calculations.

The computational model was solved using 500 iterations to ensure the full convergence of the flow parameters (Fig. 11). For analytical purposes, the measurement planes were positioned at a height of 1.1 m above the floor level (Fig. 12), corresponding to the typical human breathing zone in residential spaces. This standardized measurement approach allowed for a consistent comparison between different configuration scenarios and an accurate assessment of ventilation effectiveness for occupant comfort.

### Validation approach

**Fig. 13:**
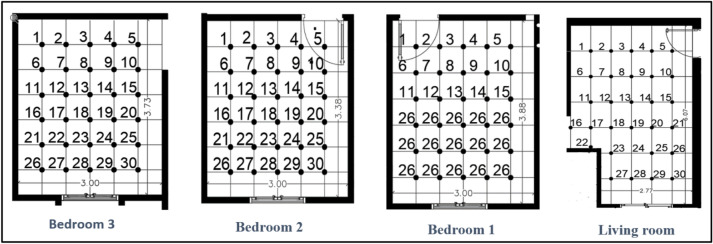
Measuring points locations.

To establish the reliability of the computational approach, a comprehensive validation study was conducted by comparing the measured site data with the simulation results. Wind speed values were collected at 30 points within each room (Fig. 13), and these measurements were directly compared with the simulation predictions at identical spatial locations. This validation process ensured that the CFD methodology could accurately predict airflow patterns and ventilation rates within residential spaces before proceeding with the analysis of different windcatcher configurations. The validation metrics included the following:

1. A point-by-point comparison of measured versus simulated velocities
2. Average velocity values for each room
3. Analysis of velocity distribution patterns within spaces
4. Egyptian standards for ventilation in buildings: In areas with a hot, humid climate, wind speed should not be less than 2 m/s, while in areas with a hot, dry climate, it should not be less than 1 m/s^35^

## Results

### Comparison between site measurement and simulation measurement

To validate the CFD methodology, a comprehensive comparison was conducted between field measurements and simulation results for the social housing building under study. Wind speed measurements were systematically collected by dividing each room into a grid with 30 sampling points (Fig. 13), creating a detailed spatial distribution of indoor airflow patterns.

Table 4 presents a comparative analysis between the site measurements and simulation results. The data revealed a close agreement between the measured and predicted values across all residential spaces. In the living room, the average measured wind speed was 48.34 cm/s, compared with the simulated value of 47.5 cm/s, representing a deviation of only 1.7%. Similar correlations were observed in other spaces: Bedroom 1 showed 34.43 cm/s (measured) versus 33.28 cm/s (simulated), Bedroom 2 exhibited 28.49 cm/s (measured) versus 29.54 cm/s (simulated), and Bedroom 3 recorded 19.32 cm/s (measured) versus 18.08 cm/s (simulated). The spatial distribution of wind velocities demonstrated consistent patterns between field observations and simulation predictions. Higher velocities were consistently recorded near the ventilation openings in both datasets, whereas lower velocities were observed in the corner zones and areas with furniture obstructions. This consistency in both the absolute values and distribution patterns confirms the validity of the CFD approach employed in this study. Statistical analysis of the data indicated that the simulation results maintained an average deviation of less than 6.5% from the field measurements across all sampling points, which is within the acceptable tolerance limits for architectural ventilation studies. The validation confirmed that the selected turbulence model and boundary conditions appropriately represented the physical airflow phenomena within the building under actual environmental conditions. The close agreement between the measured and simulated values confirmed that the computational model accurately captured the physical ventilation processes within the social housing units, thereby validating the subsequent design evaluations and recommendations.

**Table 4:** Site and simulation measurements.

Space	Site measurement (cm/s)					Average	Simulation measurement (cm/s)	Average
Living room	32	42.3	45.4	48.9	43.21	48.34		47.5
	51.8	48.7	41.2	42	42			
	38.4	52.3	49.12	42	39.17			
	38.14	57	52	61.4	43.2			
	44	48	53.2	49	47			
	42.7	66.1	63,2	56.42	43.7			
Bedroom 1	24	26.1	28	29.1	23.7	34.43		33.28
	26.1	29.4	31.2	32.54	29.1			
	29.1	31.3	23	28.1	28.4			
	31	36	38	34	30.6			
	35.13	38	39.2	38.4	34.1			
	39.3	48.6	51.2	45.3	38			
Bedroom 2	23.1	24.4	26.3	28.2	29.5	28.49		29.54
	25.9	26.2	27.72	28.4	26.3			
	26.3	25.2	25.3	27	24.12			
	26.3	26.4	27.5	28.35	26.1			
	28.3	32.4	34.7	35.15	34.4			
	30	36.5	41.2	42.1	39.47			
Bedroom 3	14.3	14.5	14.3	15.7	16.1	19.32		18.08
	15.2	18.4	21.2	19.3	19.63			
	15.76	18.3	19.3	18.2	17.3			
	15.4	18.1	20.1	18.2	16.5			
	16.7	18.3	21.5	20.51	19,43			
	17.8	21.92	23.1	24.4	21.2			

### Natural ventilation analysis of the northwest orientation apartment

**Fig. 14:**
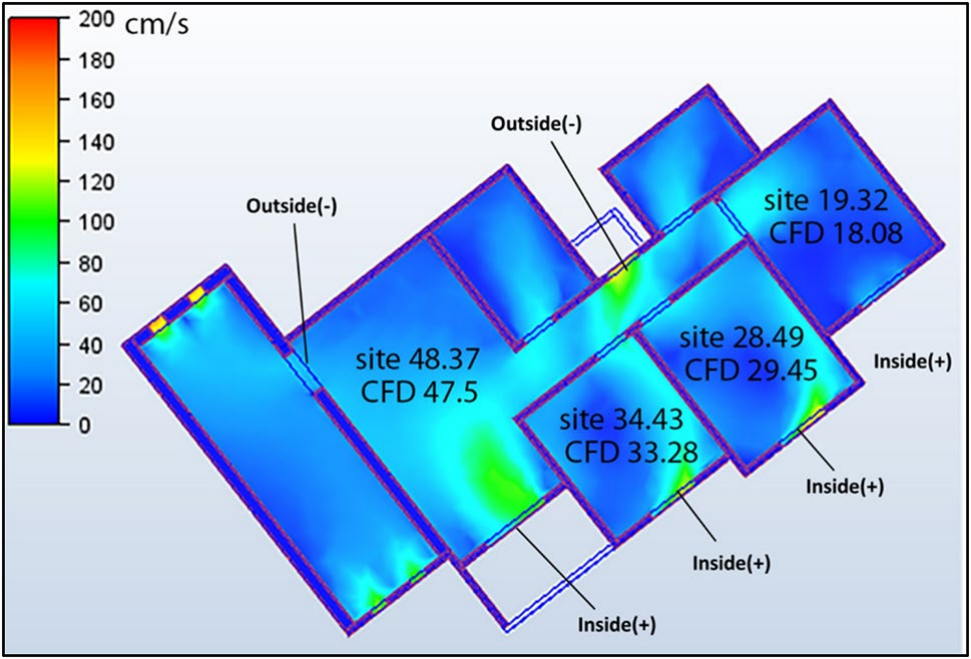
The CFD contour plot of the cross-sectional plan shows the locations of high and low wind pressures, and air velocity in the model, generated using Autodesk CFD 2019.

A comprehensive CFD analysis of the first apartment model, which had a northwest orientation, revealed significant findings regarding the windcatcher performance under various configurations. Table 5 presents the simulation results for different windcatcher integration arrangements, with velocity measurements reported in centimeters per second (cm/s) for each room in the apartment. The natural ventilation mechanics in this model predominantly relied on pressure differential dynamics, with airflow moving from high-pressure zones ( +) to low-pressure zones ( −). In this northwest-oriented apartment, the high-pressure area was consistently located at the main façade, whereas the low-pressure zones were positioned along the southwest-facing rear section of the apartment. A critical ventilation pathway was identified through the saw hole opening situated between the bathroom and kitchen, which served as the primary outlet for air circulation. The baseline model (without windcatchers) exhibited an average velocity of 30.0 cm/s throughout the apartment (Fig. 14), with the living room demonstrating the highest ventilation rate of 47.5 cm/s (Table 5). This living room focus is particularly significant, as it represents the primary family gathering space with extended occupancy periods.

**Table 5:** Simulation results of the first apartment (Northwest Orientation).

Air Speed cm/s	Model	Case1
Living room	47.5	45.64
Bedroom 1	33.28	32.36
Bedroom 2	29.54	24.68
Bedroom 3	18.08	35.46
Kitchen	21.6	18.08

Case2		Case3
Living room	45.23	49.9
Bedroom 1	31.8	35.73
Bedroom 2	25.53	31.65
Bedroom 3	33.14	21.1
Kitchen	24.9	22.93

#### Case 1: dual windcatcher configuration

**Fig. 15:**
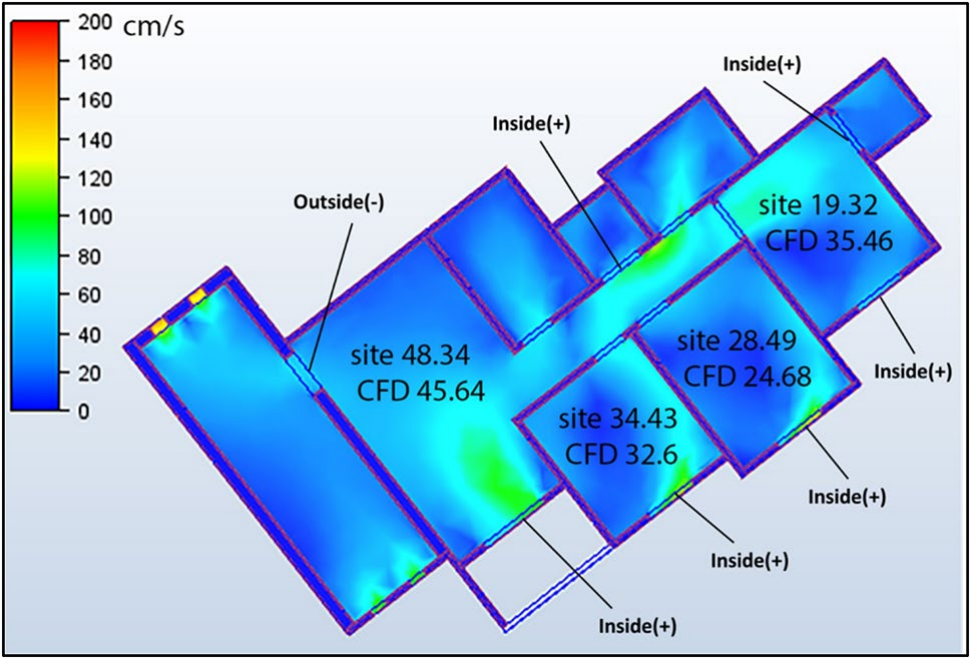
CFD contour plot of the cross-sectional plan shows the locations of high and low wind pressures and air velocity in Case 1, generated using Autodesk CFD 2019.

In the first experimental configuration (Case 1, Fig. 15), two windcatchers were strategically positioned, one at balcony 1 and the other in bedroom 3. This arrangement resulted in an unexpected decrease in the living room ventilation from 47.5 to 45.64 cm/s. This counterintuitive outcome can be attributed to the placement of windcatchers without sufficient outlet capacity, which creates a partial pressure stagnation effect. The windcatcher positioned at the saw hole location, which previously functioned as the main outlet, altered the apartment’s pressure distribution patterns, disrupting the established airflow channels. Despite this localized decrease, the overall apartment average velocity increased marginally from 30.0 to 31.44 cm/s. This improvement was predominantly driven by enhanced ventilation in bedroom 3, where the rates increased substantially from 18.08 to 35.46 cm/s owing to direct windcatcher integration. However, this improvement came at the expense of reduced ventilation rates in all other rooms, highlighting the complex interdependence of airflow dynamics within connected spaces.

#### Case 2: single windcatcher at balcony

**Fig. 16:**
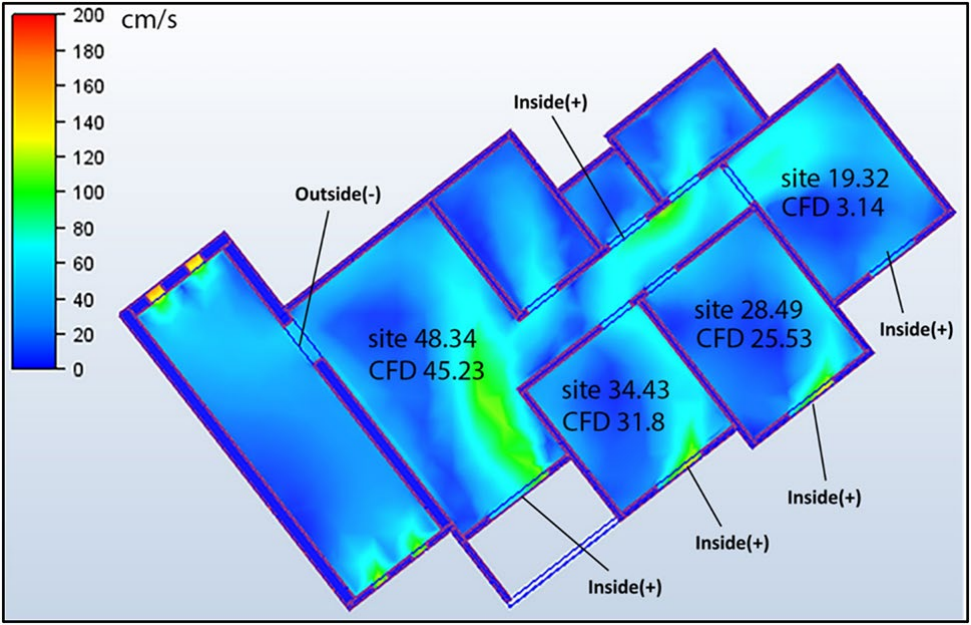
CFD contour plot of a cross-sectional plan showing the locations of high and low wind pressures and air velocity in Case 2, generated using Autodesk CFD 2019.

The second configuration (Case 2, Fig. 16) featured a single windcatcher positioned at balcony 1. This arrangement reduced the living room ventilation to 45.23 cm/s (Table 4), a consequence of transforming what had previously been a low-pressure zone into a high-pressure area through windcatcher introduction. This pressure distribution reversal reduces the pressure differential driving airflow through the living space. Interestingly, this configuration increased the apartment’s overall average ventilation to 32.12 cm/s, with bedroom 3 showing a particularly significant improvement at 33.14 cm/s. This enhancement can be explained by the geographic position of bedroom 3 in the northwest extremity of the apartment, with bedroom 2 extending beyond it. This positioning creates a natural low-pressure zone, drawing airflow from the high-pressure area generated by the wind catcher in balcony 1. The resulting pressure gradient established a defined airflow channel from the windcatcher to bedroom 3.

#### Case 3: single windcatcher in bedroom

**Fig. 17:**
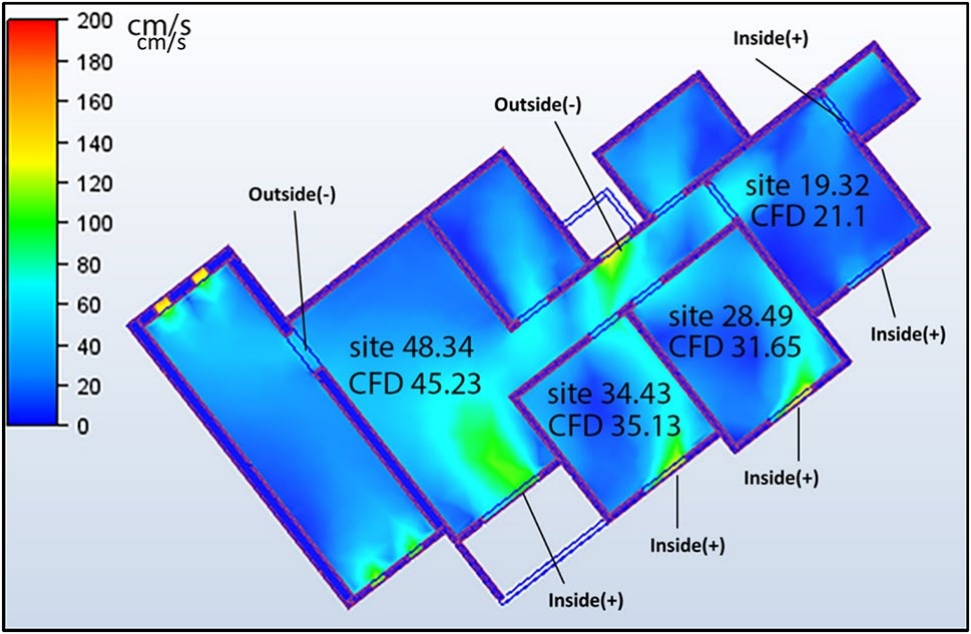
Shows the CFD contour plot of the cross-sectional plane, which indicates the locations of high and low wind pressures and air velocity in Case 3, generated using Autodesk CFD 2019.

**Fig. 18:**
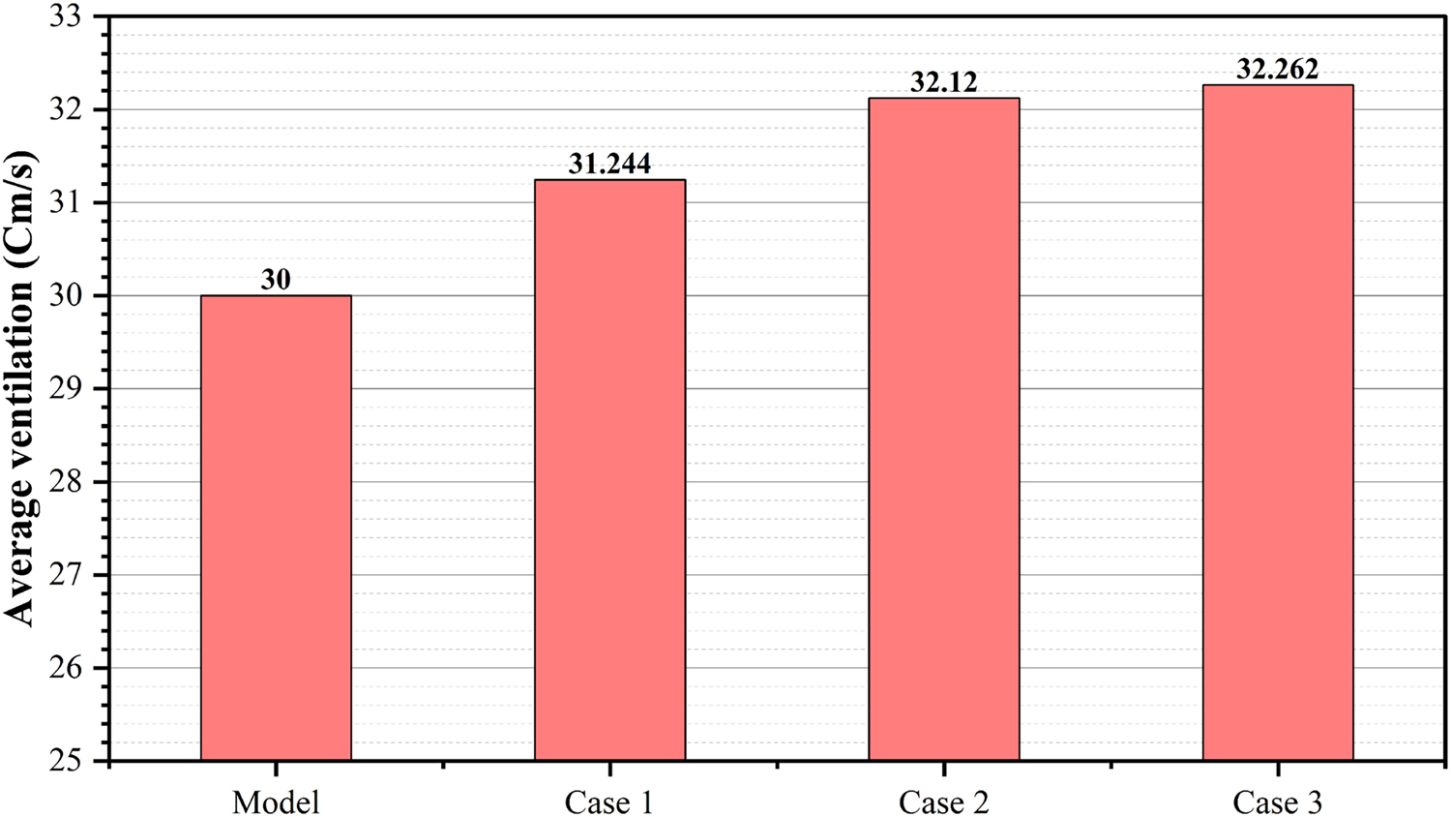
Average ventilation for each apartment under different conditions.

The third configuration (Case 3, Fig. 17) implemented a single windcatcher in bedroom 3, yielding the most favorable results among all the tested arrangements. This configuration increased the living room ventilation to 49.9 cm/s (Table 4) and raised the apartment’s overall average velocity to 32.62 cm/s. The superior performance of this configuration can be attributed to the optimization of the natural ventilation pathways. By positioning the windcatcher in bedroom 3 while maintaining balcony 1 as an undisturbed low-pressure outlet zone, this arrangement facilitated more effective cross-ventilation throughout the apartment. The presence of clearly defined inlet and outlet points created a consistent airflow path, enhancing the ventilation efficiency in both the windcatcher-equipped bedroom and adjacent spaces, including the living room. Figure 18 shows the deference in average ventilation for each case.

**Fig. 19:**
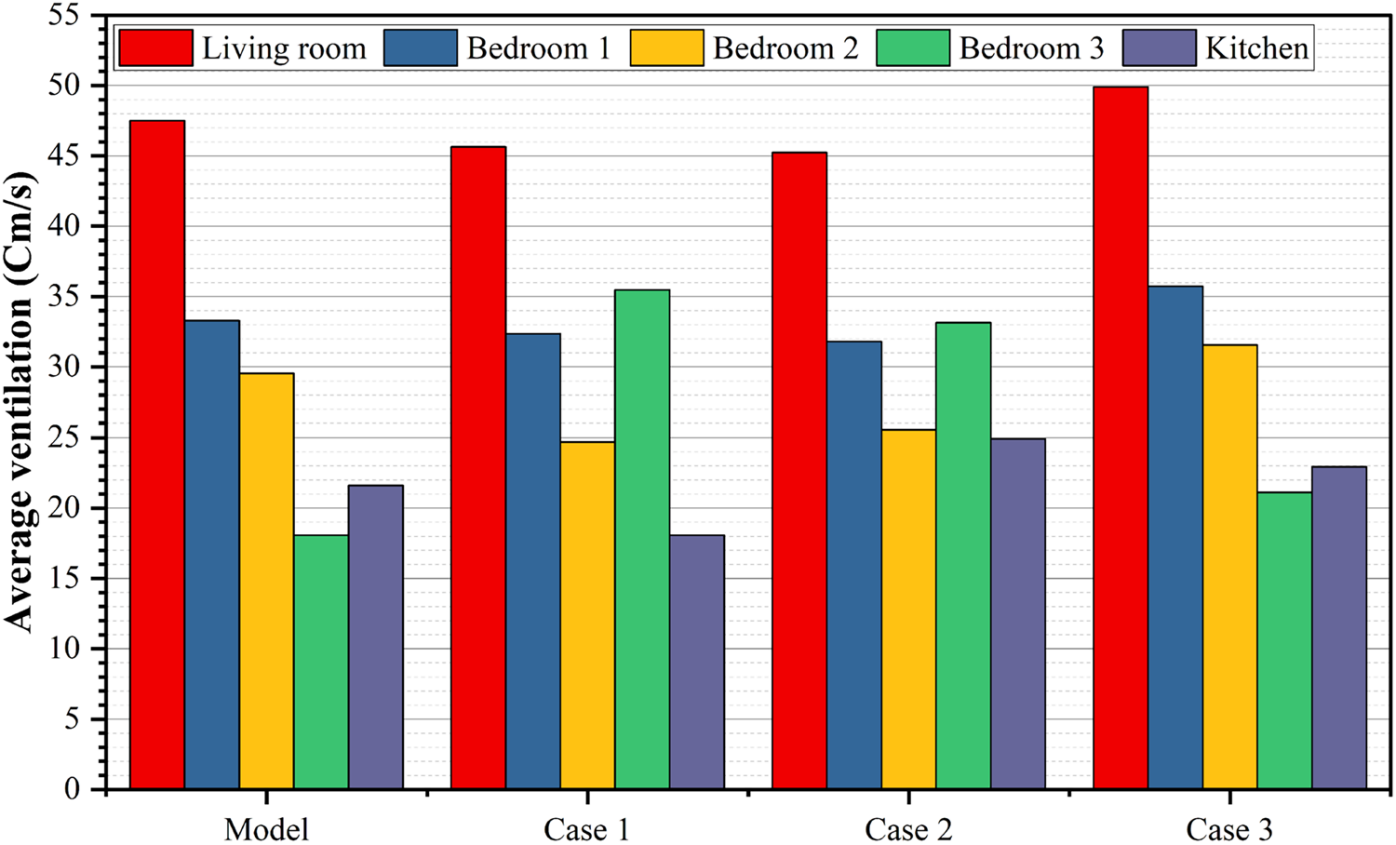
Average ventilation for each room in different cases.

Figure 19 provides a comprehensive visualization of the simulation results for all rooms in the apartment under different windcatcher configurations. This comparative analysis demonstrates that Case 3, with its single windcatcher in bedroom 3, produced the most balanced and effective ventilation enhancement throughout the apartment. This configuration not only improved thermal comfort in the primary living spaces but also maintained adequate ventilation in the peripheral rooms, creating a more uniformly comfortable indoor environment. From the previous 3 proposed designs for windcatcher integration, we noticed that the natural ventilation rates improved by 7% with a single windcatcher positioned strategically in bedroom 3.

### Natural ventilation analysis of the southwest-oriented apartment

**Fig. 20:**
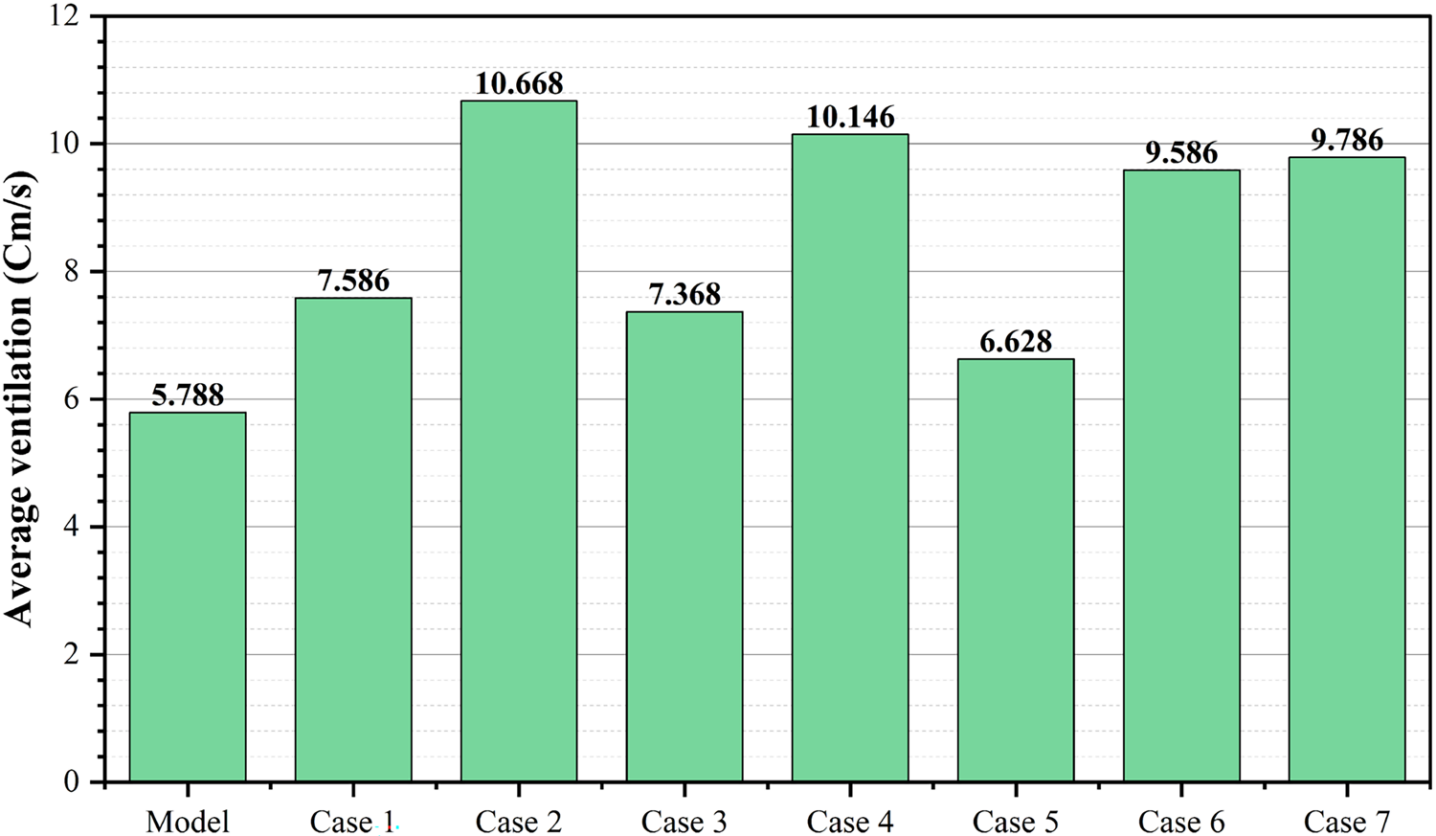
Average(cm/s) ventilation for each apartment under different situations.

**Fig. 21:**
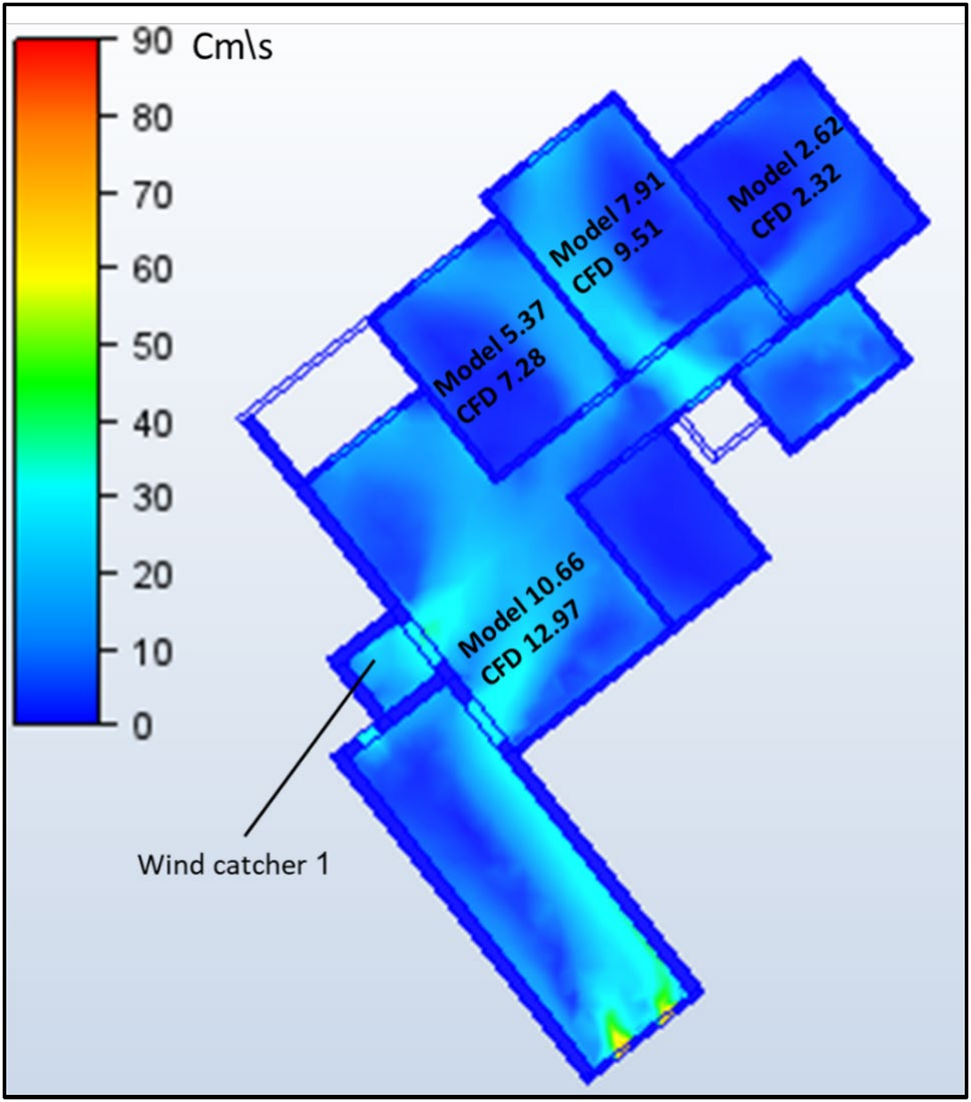
CFD contour plot of a cross-section showing the effect of the wind catcher on the ventilation process inside the apartment in Case 3*, generated using Autodesk CFD 2019*.

The second model under investigation comprised an apartment with a southwest orientation. Table 6 presents the comprehensive simulation results for the apartment, displaying the velocity measurements (cm/s) across various windcatcher configurations. The baseline model without windcatcher integration exhibited an average velocity of 5.788 cm/s throughout the apartment spaces (Fig. 20), with the living room demonstrating the highest natural ventilation rate at 10.66 cm/s. This represents a significant baseline reduction compared to the northwest-oriented apartment analyzed in Sect. 2.5, primarily attributable to the less favorable orientation relative to the prevailing wind patterns (Fig. 21).

**Table 6:** Simulation results for the second apartment (Southwest orientation).

Air Speed cm/s	Model	Case1	Case2	Case3
Living room	10.66	12.61	14.1	12.97
Bedroom 1	5.37	6.74	9.24	7.28
Bedroom 2	7.91	10.12	11.13	9.51
Bedroom 3	2.62	4.73	14.62	4.76
Kitchen	2.38	3.73	4.25	2.32

Air Speed cm/s	Case4	Case5	Case6	Case7
Living room	13.81	10.56	12.64	12.26
Bedroom 1	8.56	6.26	7.1	8.05
Bedroom 2	10.53	8.52	9.94	9.94
Bedroom 3	14.73	1.74	14.72	14.21
Kitchen	3.1	4.26	3.53	4.92

#### Single windcatcher configurations (cases 3, 5, and 6)

**Fig. 22:**
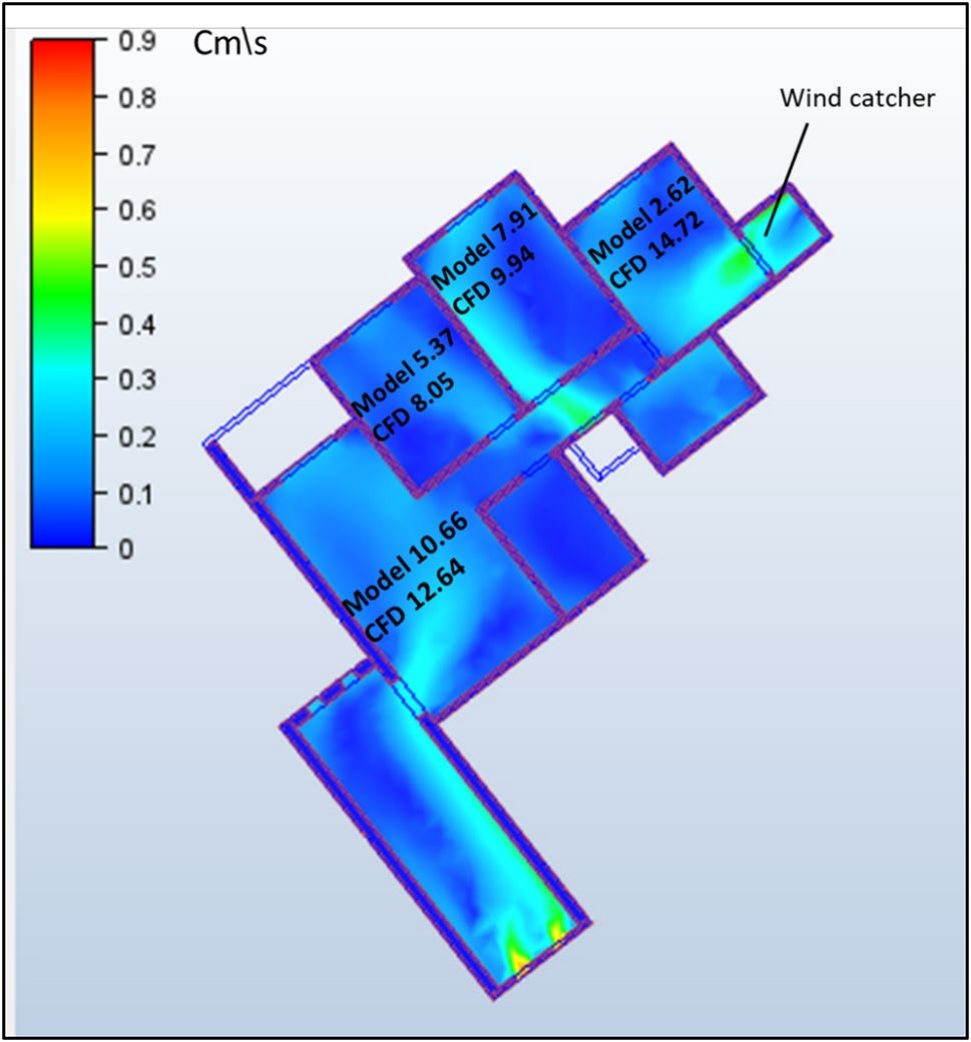
CFD contour plot of a cross-section showing the effect of the wind catcher on the ventilation process inside the apartment in Case 6*, generated using Autodesk CFD 2019*.

**Fig. 23:**
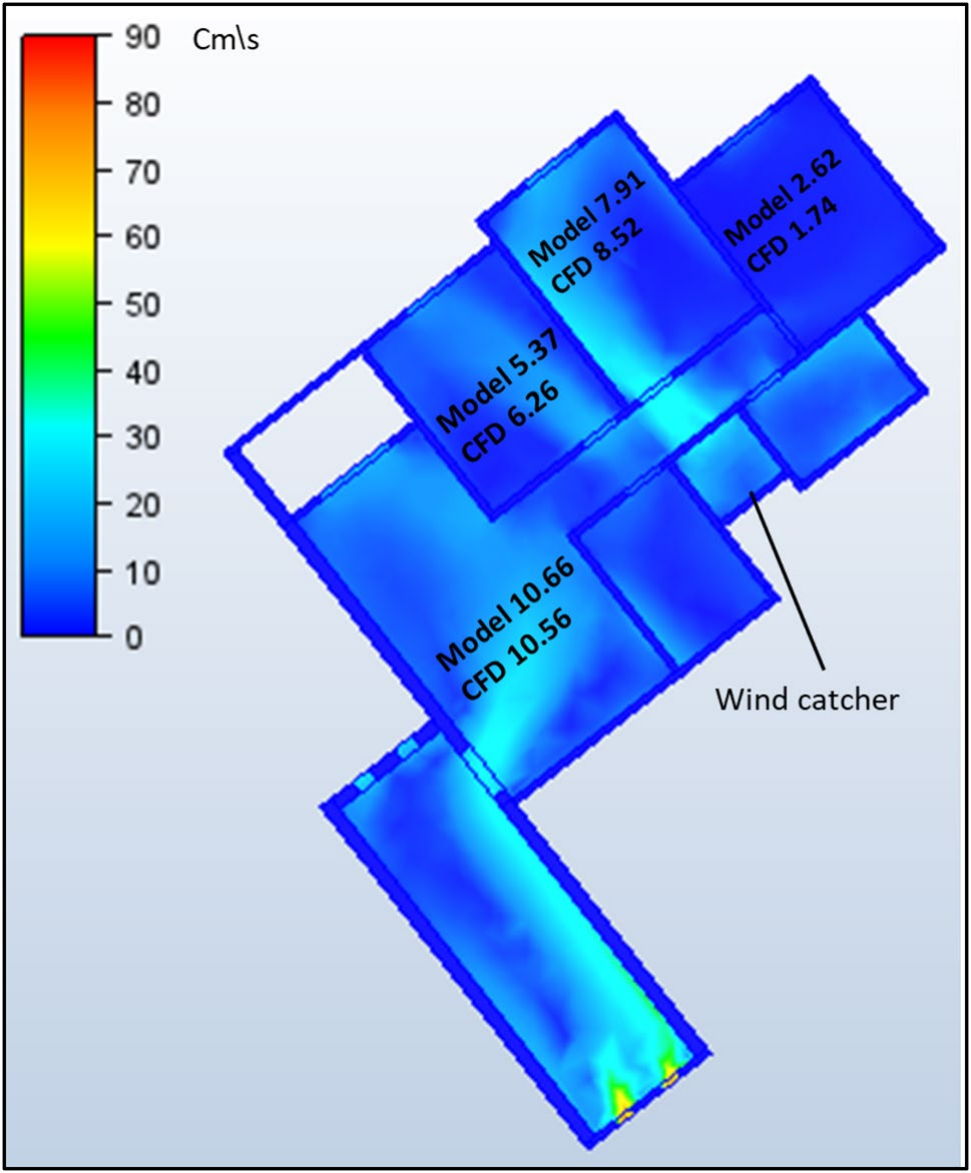
CFD contour plot of a cross-section showing the effect of the wind catcher on the ventilation process inside the apartment in Case 5*, generated using Autodesk CFD 2019*.

The implementation of a single windcatcher in Cases 3, 5, and 6 yielded measurable improvements in the ventilation rates. In Case 3, positioning the windcatcher in the living room increased the average ventilation of the apartment to 7.368 cm/s (Fig. 20), with the living room velocity rising to 12.97 cm/s (Table 6). This configuration proved particularly effective as the windcatcher’s placement in the living room, which faces the primary street frontage, created an efficient air circulation pathway that enhanced ventilation throughout the connected bedroom spaces (Fig. 22). The introduction of high-pressure zones at the windcatcher inlet generated a pressure differential that facilitated cross-ventilation through the interior spaces of the apartment. Case 6 demonstrated superior performance among the single windcatcher arrangements, with the average ventilation rate increasing substantially to 9.586 cm/s (Fig. 20). This configuration, featuring a windcatcher positioned in bedroom 3, achieved a living room velocity of 12.64 cm/s while simultaneously enhancing ventilation across all residential spaces (Table 5). The notable improvement resulted from the strategic positioning that established an effective airflow pathway between the living room and bedroom 3 (Fig. 21), creating a continuous ventilation circuit that maximized air exchange. Conversely, Case 5 yielded the lowest ventilation performance, with an average velocity of 6.268 cm/s (Fig. 20) and a living room velocity of 10.56 cm/s (Table 6). This suboptimal outcome can be attributed to the windcatcher’s location at balcony 1, facing bedrooms 2 and 3, which improved ventilation exclusively in those spaces while adversely affecting airflow patterns in other rooms (Fig. 23). The localized enhancement failed to establish effective whole-apartment ventilation.

#### Dual windcatcher configurations (cases 1, 4, and 7)

**Fig. 24:**
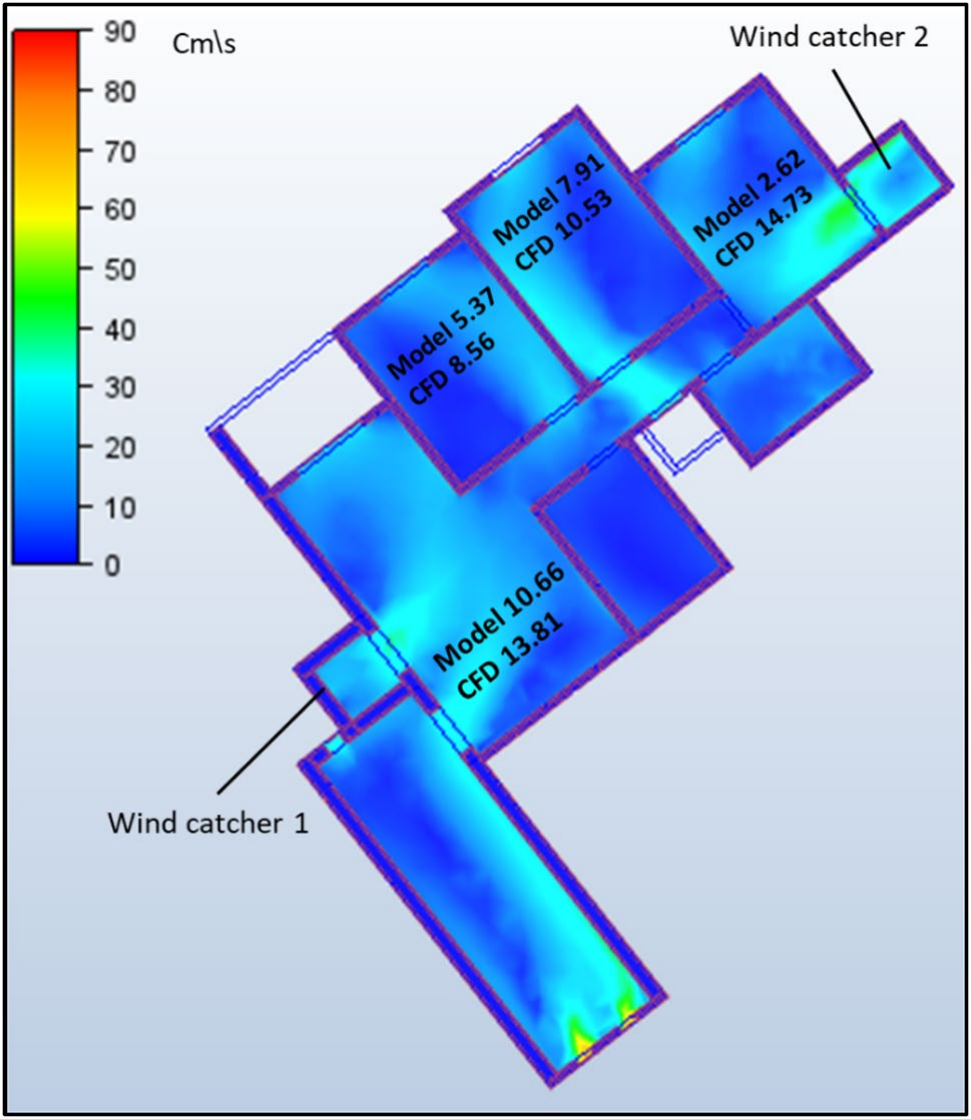
CFD contour plot of a cross-section showing the effect of the wind catcher on the ventilation process inside the apartment in Case 4*, generated using Autodesk CFD 2019*.

**Fig. 25:**
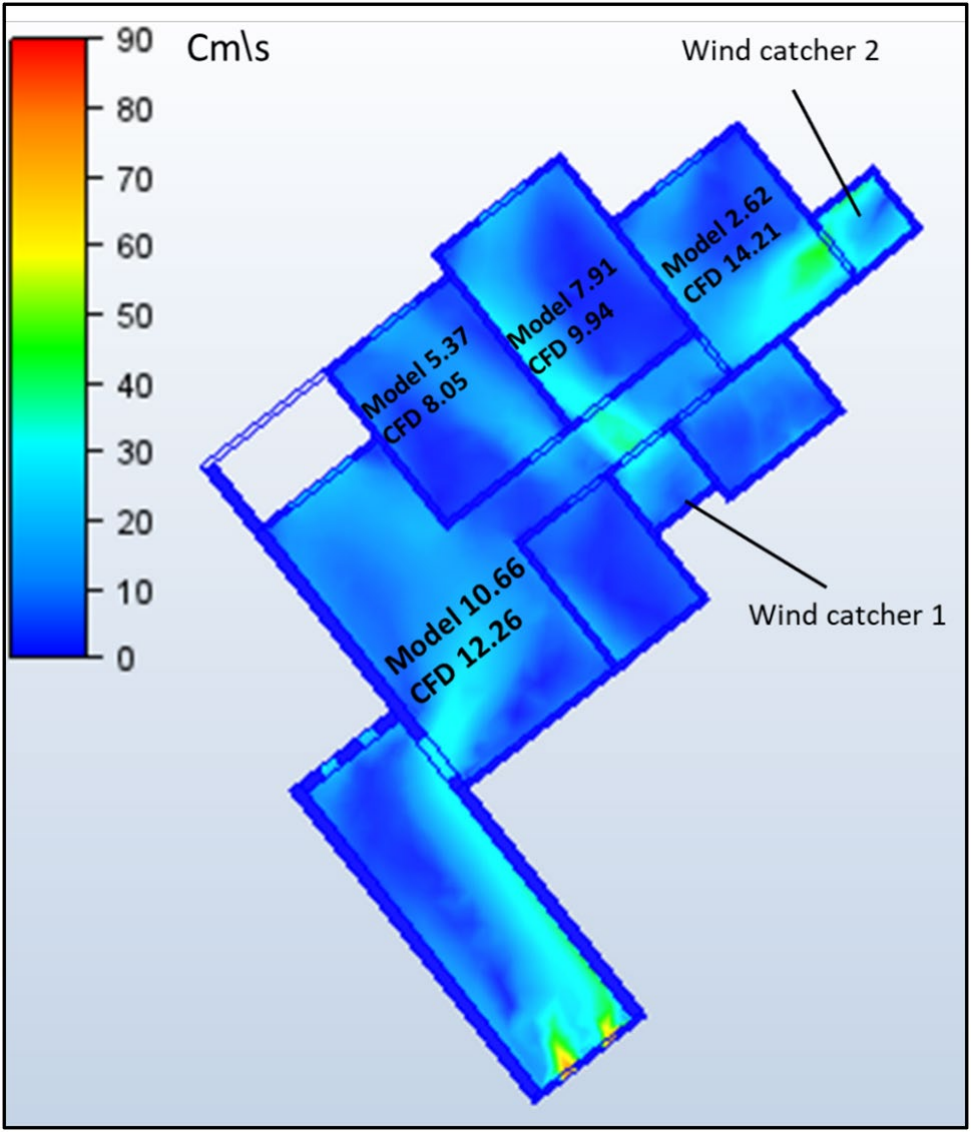
CFD contour plot of a cross-section showing the effect of the wind catcher on the ventilation process inside the apartment in Case 7*, generated using Autodesk CFD 2019*.

**Fig. 26:**
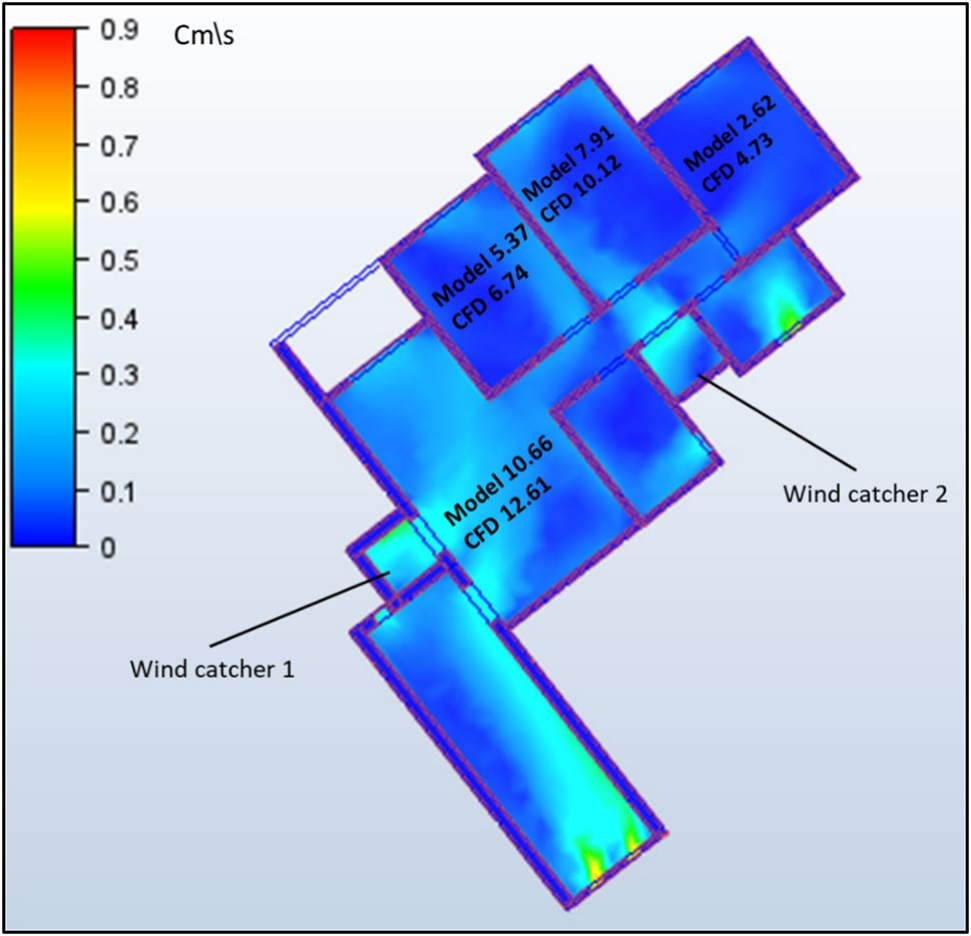
CFD contour plot of a cross-section showing the effect of the wind catcher on the ventilation process inside the apartment in Case 1*, generated using Autodesk CFD 2019*.

The implementation of two windcatchers significantly enhanced the ventilation performance across multiple configurations. Case 4 demonstrated exceptional effectiveness, with an average velocity of 10.146 cm/s (Fig. 20) and a living room velocity of 13.81 cm/s (Table 6). The strategic positioning established a well-defined pressure gradient that facilitated consistent airflow throughout the apartment interior (Fig. 24), with the two windcatchers effectively working in tandem to maximize ventilation efficiency. Case 7, with windcatchers positioned in balconies 1 and 3, achieved an average ventilation rate of 9.876 cm/s (Fig. 20) and a living room velocity of 12.26 cm/s (Table 5). This configuration prioritized bedroom ventilation, with the balcony 1 windcatcher serving bedrooms 1 and 2, whereas the second windcatcher directly enhanced bedroom 3 (Fig. 25). The resulting distributed ventilation pattern improved the overall air circulation throughout the apartment. In contrast, Case 1 yielded the lowest performance among the dual-windcatcher configurations, with an average velocity of 7.586 cm/s (Fig. 20) and a living room velocity of 12.61 cm/s (Table 6). This arrangement placed windcatchers in the living room and balcony 1, effectively concentrating ventilation enhancement in the apartment’s front section while providing minimal benefit to bedroom 3 (Fig. 26). The imbalanced distribution created ventilation dead zones that reduced the overall efficiency.

#### Triple windcatcher configuration (case 2)

**Fig. 27:**
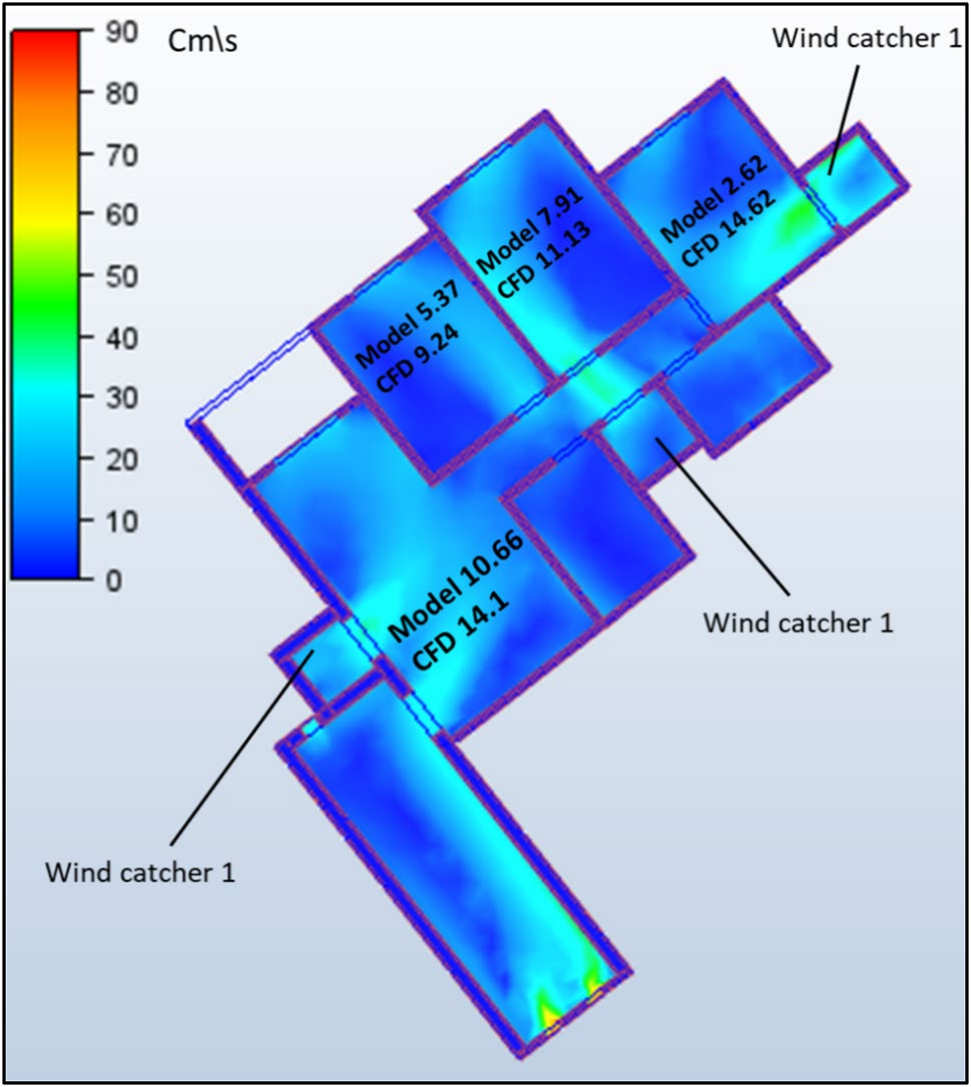
CFD contour plot of a cross-section showing the effect of the wind catcher on the ventilation process inside the apartment in Case 2*, generated using Autodesk CFD 2019*.

**Fig. 28:**
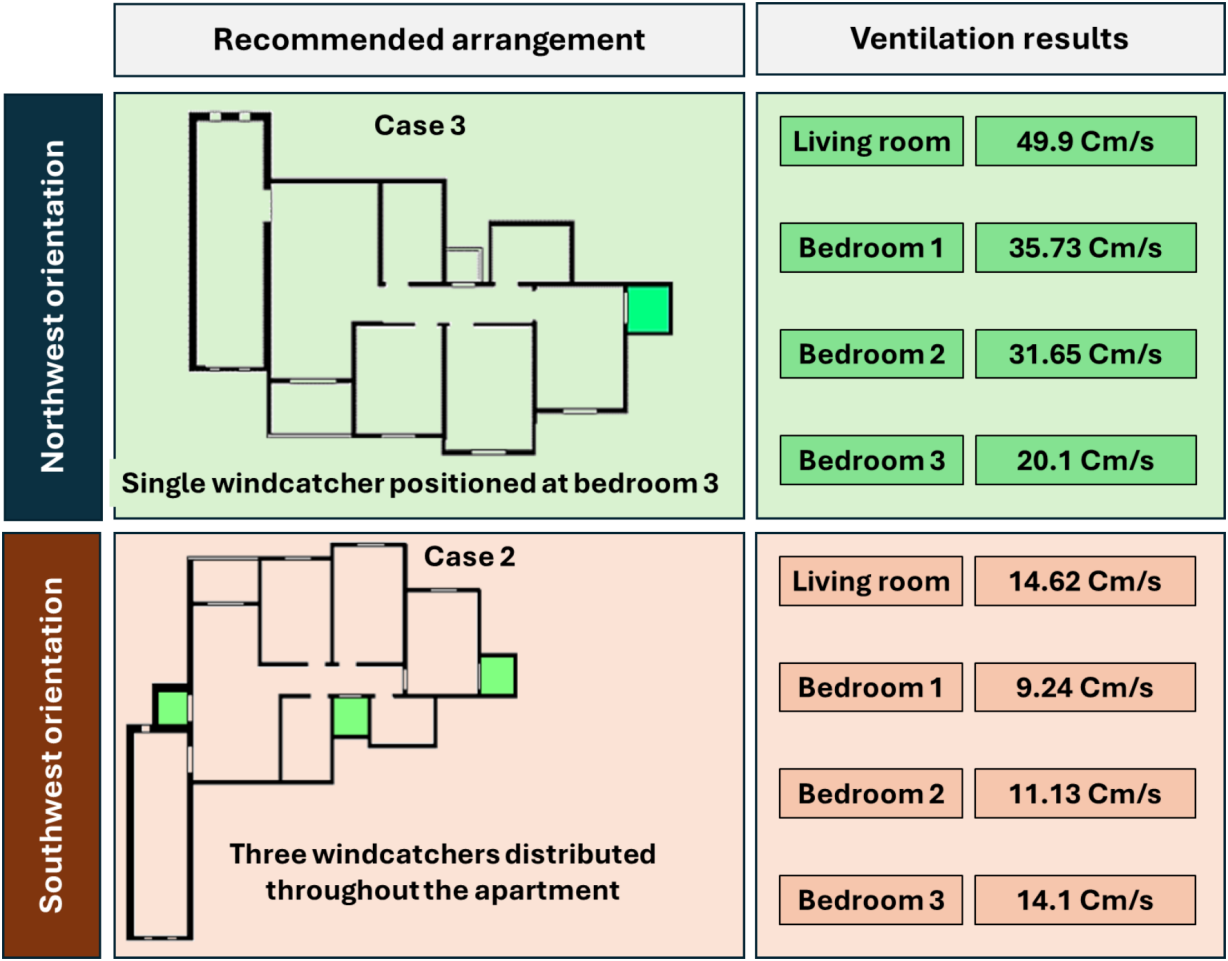
Optimum arrangement and its corresponding ventilation of applied windcatcher for the northwest and southwest directions.

**Fig. 29:**
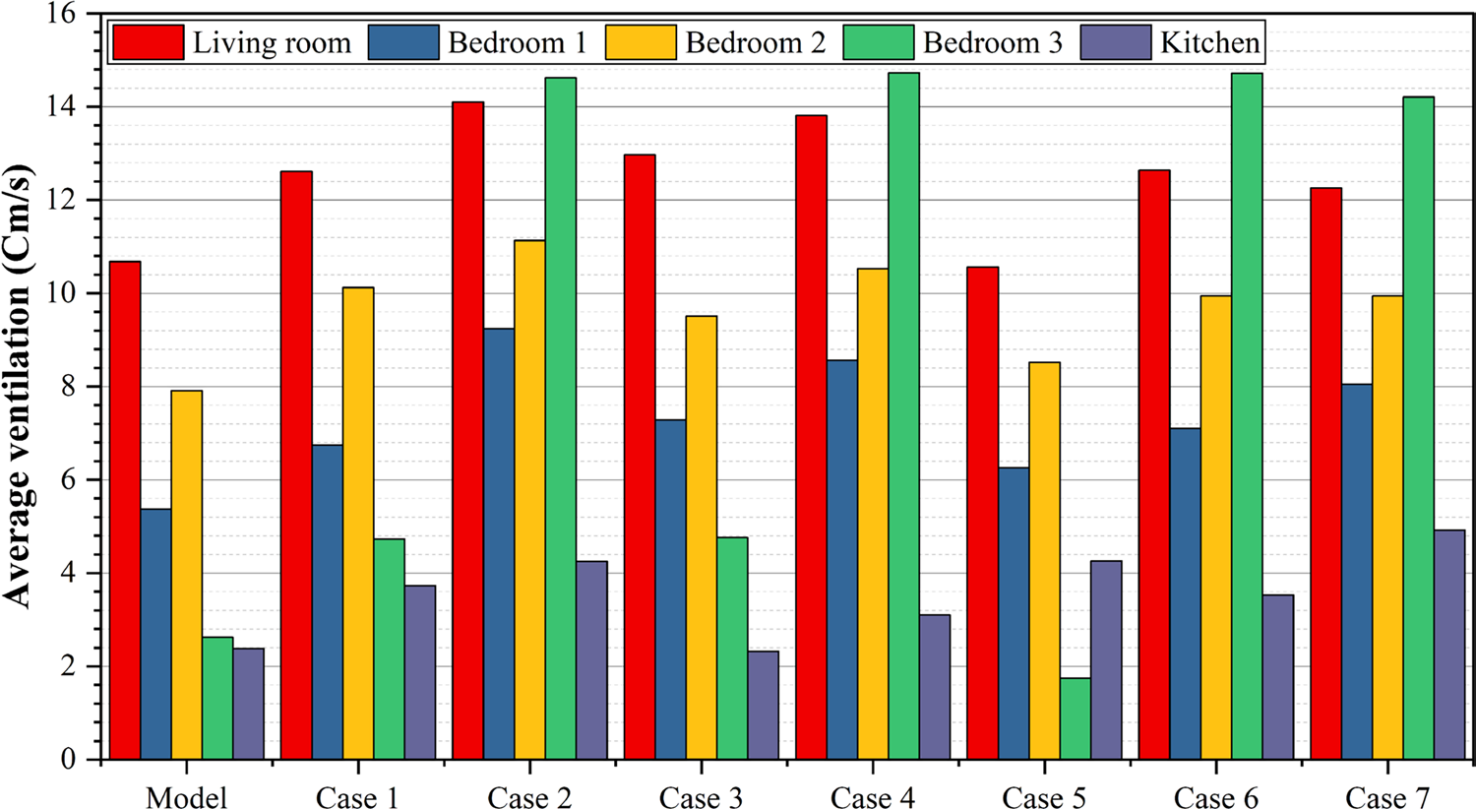
Average ventilation for each room in the different cases.

The most comprehensive ventilation enhancement was achieved in Case 2, which implemented three windcatchers strategically distributed throughout the apartment. This configuration yielded the highest overall ventilation rate of 10.668 cm/s (Fig. 20) and a living room velocity of 14.1 cm/s (Table 6). Windcatchers positioned in the living room, balcony 1, and bedroom 3 established multiple coordinated inlet zones that created a distributed pressure network throughout the apartment (Fig. 27). This arrangement eliminated ventilation dead zones by providing direct air inlets to all major residential spaces, resulting in uniform and efficient ventilation throughout the apartment unit (Fig. 28). From the previous proposed designs for windcatcher integration, we noticed that the natural ventilation rates improved by 45.7% when equipped with three windcatchers distributed across the living room and bedroom areas, also we can notice that either a triple-windcatcher system (living room, balcony, and bedroom 3) or a dual-windcatcher system (living room and bedroom 3) is recommended, depending on construction budget constraints, as shown in Fig. 29.

#### Comparative analysis

**Fig. 30:**
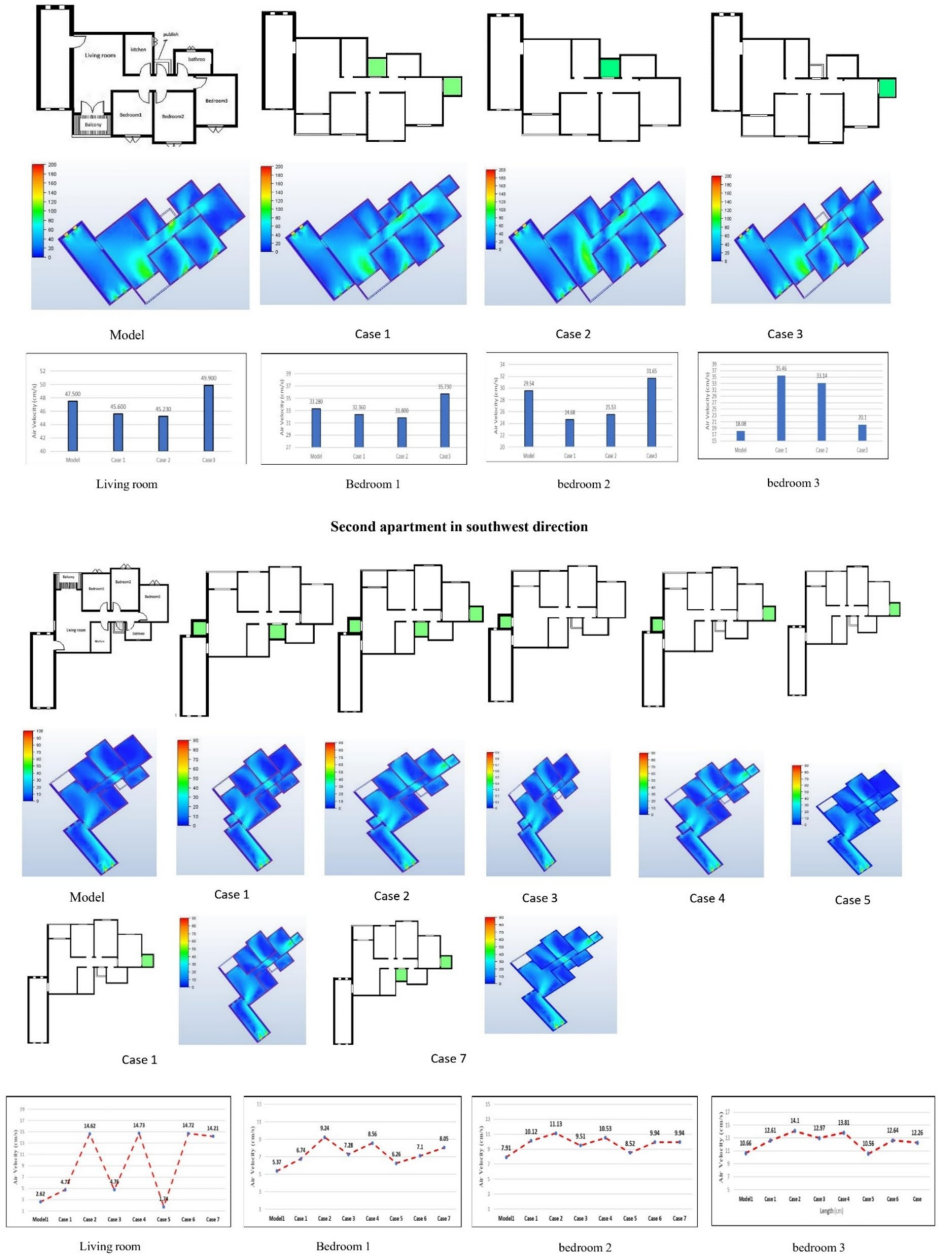
Detailed comparison of the ventilation rates across all the rooms for all proposed windcatcher integration for both apartments.

Figure 28 shows a detailed comparison of the ventilation rates across all the rooms under different windcatcher configurations, while (Fig. 29) shows the Optimum arrangement and its corresponding ventilation of the applied windcatcher for the northwest and southwest orientations. The data demonstrate that Cases 2 (triple windcatcher) and 4 (dual windcatcher) achieved the most significant ventilation enhancement, with improvements of 45.7% and 43.2%, respectively, compared to the baseline model. These findings indicate that strategic windcatcher placement is more critical than mere quantity, as evidenced by the comparable performance of Case 4 to that of Case 2, despite the former utilizing fewer windcatchers. The results further demonstrate that orientation significantly influences windcatcher effectiveness, with the southwest-oriented apartment experiencing substantially lower baseline ventilation compared with the northwest-oriented model analyzed in Sect. 2.5. This orientation-dependent performance underscores the importance of adapting windcatcher configurations to specific building orientations to maximize the benefits of natural ventilation in social housing designs. Figure 30 shows a detailed comparison of the ventilation rates across all the rooms for all proposed windcatcher integration for both apartments.

## Conclusion, recommendations, and future directions

### Conclusion

This study conducted numerical and field data to investigate the effectiveness of integrating windcatchers into Egyptian social housing buildings to enhance natural ventilation rates and indoor thermal comfort, providing validated design guidance to develop the design outlines for enhancing energy consumption reduction. The windcatcher placement and quantity have been investigated at various residential units oriented differently (northwest and southwest). This was achieved through rigorous CFD simulations, validated by field measurements, and compared with Egyptian standards for ventilation in residential buildings. Using the integrated windcatcher in Egypt’s social housing helps improve and increase ventilation rates inside the buildings’ architectural spaces. The finding refers to optimizing the rates to be aligned with the Egyptian standards rates of ventilation according to Egyptian standards for ventilation in buildings^26^. The investigation of two distinctly oriented apartment models yielded several important conclusions:

- Natural ventilation rates improved by 7% in the northwest-oriented apartment with a single windcatcher positioned strategically in bedroom 3, whereas the southwest-oriented apartment demonstrated a remarkable 45.7% improvement when equipped with three windcatchers distributed across the living room and bedroom areas.
- The results revealed that the strategic placement of windcatchers was more critical than their number. In the southwest-oriented apartment, the dual-windcatcher configuration (Case 4) achieved a performance nearly comparable (43.2% improvement) to that of the triple-windcatcher arrangement (45.7% improvement), demonstrating that optimal positioning can maximize efficiency while minimizing construction costs.
- The study identified that the effectiveness of windcatchers relies heavily on establishing appropriate pressure gradients. Careful positioning is essential for creating clearly defined high- and low-pressure zones that facilitate consistent airflow circulation throughout residential spaces.
- The configurations that enhanced ventilation in the living room, the primary family gathering space with extended occupancy periods, demonstrated the greatest potential for improving the overall thermal comfort and occupant well-being.

For the proposed designs upon the findings of the current research, the study focused on the windcatcher placement and quantity to observe its impact on the achieved fresh air rates. Ideally, the proposed designs, which require a smaller space for integration and lower cost, are preferred. However, it should still be able to prevent the air short-circuiting of the fresh air into the building. Hence, a detailed evaluation of ventilation performance is required before progressing with using the developed proposals. The future research can focus on the effects of windcatcher geometry, slope angle, and projection length on improving the thermal performance of the windcatcher and evaluating the impact on the thermal comfort of occupants. A further wind tunnel is necessary to further validate the numerical modeling.

### Recommendation for practical applications

Based on these findings, the following practical recommendations are proposed for implementing windcatchers in Egyptian social housing:

- Orientation-specific design strategies: For northwest-oriented apartments, a single windcatcher positioned in the bedroom areas (specifically, bedroom 3, as demonstrated in Case 3) provides optimal ventilation enhancement. For southwest-oriented apartments, either a triple-windcatcher system (living room, balcony, and bedroom 3) or a dual-windcatcher system (living room and bedroom 3) is recommended, depending on construction budget constraints.
- Windcatcher placement should be integrated early in the architectural design process, particularly considering the building orientation relative to prevailing winds, to maximize the benefits of natural ventilation.
- The demonstrated effectiveness suggests that standardized windcatcher designs could be incorporated into building codes and specifications for social housing projects across Egypt’s new urban communities.
- The substantial ventilation improvements achieved with minimal architectural intervention represent a cost-effective approach to enhancing indoor environmental quality in affordable housing without the need for expensive mechanical systems.
- The implementation of the program at scale across Egypt’s social housing program could generate significant cumulative energy savings.

### Future research directions

This study highlights several promising avenues for future research:

- Extended studies examining windcatcher performance across different seasons and weather conditions will provide more comprehensive design guidance.
- Investigation of windcatchers combined with other cooling and heating strategies.
- A detailed analysis of windcatcher geometry, including height, cross-section, and internal baffle design, could refine the performance characteristics for the specific climate conditions of Egypt’s different regions.

## Data Availability

The datasets used and/or analyzed during the current study are available from the corresponding author on reasonable request.
